# AIME: Autoencoder-based integrative multi-omics data embedding that allows for confounder adjustments

**DOI:** 10.1371/journal.pcbi.1009826

**Published:** 2022-01-26

**Authors:** Tianwei Yu

**Affiliations:** 1 School of Data Science, The Chinese University of Hong Kong–Shenzhen, Shenzhen, Guangdong, China; 2 Shenzhen Research Institute of Big Data, Shenzhen, Guangdong, China; 3 Warshel Institute for Computational Biology, Shenzhen, Guangdong, China; Children’s National Hospital, UNITED STATES

## Abstract

In the integrative analyses of omics data, it is often of interest to extract data representation from one data type that best reflect its relations with another data type. This task is traditionally fulfilled by linear methods such as canonical correlation analysis (CCA) and partial least squares (PLS). However, information contained in one data type pertaining to the other data type may be complex and in nonlinear form. Deep learning provides a convenient alternative to extract low-dimensional nonlinear data embedding. In addition, the deep learning setup can naturally incorporate the effects of clinical confounding factors into the integrative analysis. Here we report a deep learning setup, named Autoencoder-based Integrative Multi-omics data Embedding (AIME), to extract data representation for omics data integrative analysis. The method can adjust for confounder variables, achieve informative data embedding, rank features in terms of their contributions, and find pairs of features from the two data types that are related to each other through the data embedding. In simulation studies, the method was highly effective in the extraction of major contributing features between data types. Using two real microRNA-gene expression datasets, one with confounder variables and one without, we show that AIME excluded the influence of confounders, and extracted biologically plausible novel information. The R package based on Keras and the TensorFlow backend is available at https://github.com/tianwei-yu/AIME.

## Author summary

Integrative analysis, i.e. jointly analyzing two or more data matrices, is becoming more and more common in omics research. One type of integrative analysis measures the association between two groups of variables by finding low-dimensional spaces that maximize certain measures of agreement between the data matrices. Representative methods in this area include Canonical Correlation Analysis (CCA), Partial Least Squares (PLS), Multi-Omics Factor Analysis (MOFA), integrative clustering (iCluster), Similarity Network Fusion (SNF), joint Singular Value Decomposition (jSVD) *etc*. Here we present a new method: Autoencoder-based Integrative Multi-omics data Embedding (AIME). The method jointly analyzes two data matrices. It finds data embedding from the input data matrix that best preserves its relation with the output data matrix. It has several characteristics: (1) It is based on neural network. Hence it can detect nonlinear associations between the data matrices; (2) It can adjust for confounding variables such as age, gender, ethnicity *etc*, to remove their effects in the low-dimensional space; (3) It estimates pairwise relations between variables in the two data matrices. It is a useful addition to the tools for integrative analysis.

This is a *PLOS Computational Biology* Methods paper.

## 1. Introduction

In more and more studies, multiple omics data are collected on the same set of subjects to obtain a global view of the molecular signature of a disease. When analyzing such data, a common task is to find data embedding in a lower dimensional space from one data type that best preserves the information pertaining to another data type. Such data embedding can reveal mechanistic relations between the data types, or serve as extracted predictors in predictive models.

To achieve data embedding while considering two data types, the most common methods are dimension reduction approaches including Canonical Correlation Analysis (CCA), Partial Least Squares (PLS) and their variants, which are based on (sparse) linear projections of the data [1–3]. Given the complexity of omics data, nonlinear equivalents to such linear methods were developed, such as kernel-based [4] and deep learning-based CCA [5]. Beside dimension reduction, factorization and clustering techniques were also developed to analyze multiple data types collected on the same set of samples. For example, iCluster and other multi-view clustering algorithms seek the co-clustering of variables from different omics data types [6,7]. Multi-Omics Factor Analysis (MOFA) and MOFA2 find data embedding by jointly modelling variation across data types [8,9]. Joint Singular Value Decomposition (jSVD), or Simultaneous Component Analysis (SCA), was used to find a single set of unitary matrices to simultaneously diagonalize multiple matrices [10]. Similarity Network Fusion (SNF) combines multi-omics data through the fusion of sample similarity network [11].

In biomedical studies, it is often of interest to extract data representation while adjusting for linear/non-linear effects of clinical confounding factors, such as age, gender, ethnicity, batch effects *etc*. Current approaches do not adjust for confounders, which could lead to data embedding and sample grouping that are heavily influenced by the confounding factors, weakening the signal from true biological relations between the data types.

Autoencoder is a deep learning–based nonlinear embedding approach that is typically used to achieve sparse data representation from a single dataset [12], reduce noise [13], impute missing values [14], conduct pre-training for classification tasks [15], and make functional inferences [16–19]. A supervised auto-encoder (SAE) is an auto-encoder with the additional supervised loss component on the representation layer, which factors in a second data type, with a goal of improving generalization performance [20]. Variants of autoencoders have been used in combining multiple data matrices. In terms of integrative analysis, joint learning schemes [21,22] are used to combine multi-omics data in both the input and reconstruction layers in order to find their interactions for the prediction task. Combining joint learning with probabilistic Gaussian Mixture Model was able to learn informative joint latent features to construct the association between omics data and find cell heterogeneity [23].

In this study, our goal is geared towards data interpretation. We aim at achieving nonlinear data embedding from one omics data type, in order to preserve the information pertaining to another omics data type. Prediction or reconstruction is not a major concern. We modify the autoencoder structure by using two data types in the input and reconstruction layers respectively, and allowing contributions from clinical confounding factors by including them as auxiliary inputs at the representation layer. The approach is different than existing joint learning methods [21–23] in that it doesn’t seek joint embedding which could be a mixture of the two data types, in which their individual contributions are hard to separate, and it doesn’t involve outcome variables. It is also different than Deep learning-based CCA [5], which tries to find two separate embeddings from the two data types that are highly correlated, and doesn’t allow the adjustment of confounders. Here our main interest is to find features in one data type that influence the other data type.

The method is named Autoencoder-based Integrative Multi-omics data Embedding (AIME). In simulations, we show the method can effectively extract influential features. When sample size is large, the method can be more sensitive than CCA and PLS even when all relations are linear. In real data analysis, the method can exclude the superficial relations caused by clinical confounding factors, and extract meaningful miRNAs that influence gene expression.

## 2. Methods

### 2.1. The setup

Assume there are two types of high-throughput measurements on the same set of samples. Let ***X***_***N***×***p***_ denote the first data type, where there are ***p*** features and ***N*** samples. Let ***Y***_***N***×***q***_ denote the second data type, where there are ***q*** features and ***N*** samples. Our interest is to extract a low-dimensional nonlinear data embedding from the ***X*** matrix, ***E***_***N***×***r***_, where ***r*** is small, such that the ***E*** matrix contains as much information to nonlinearly reconstruct the ***Y*** matrix as possible.

**Fig 1:**
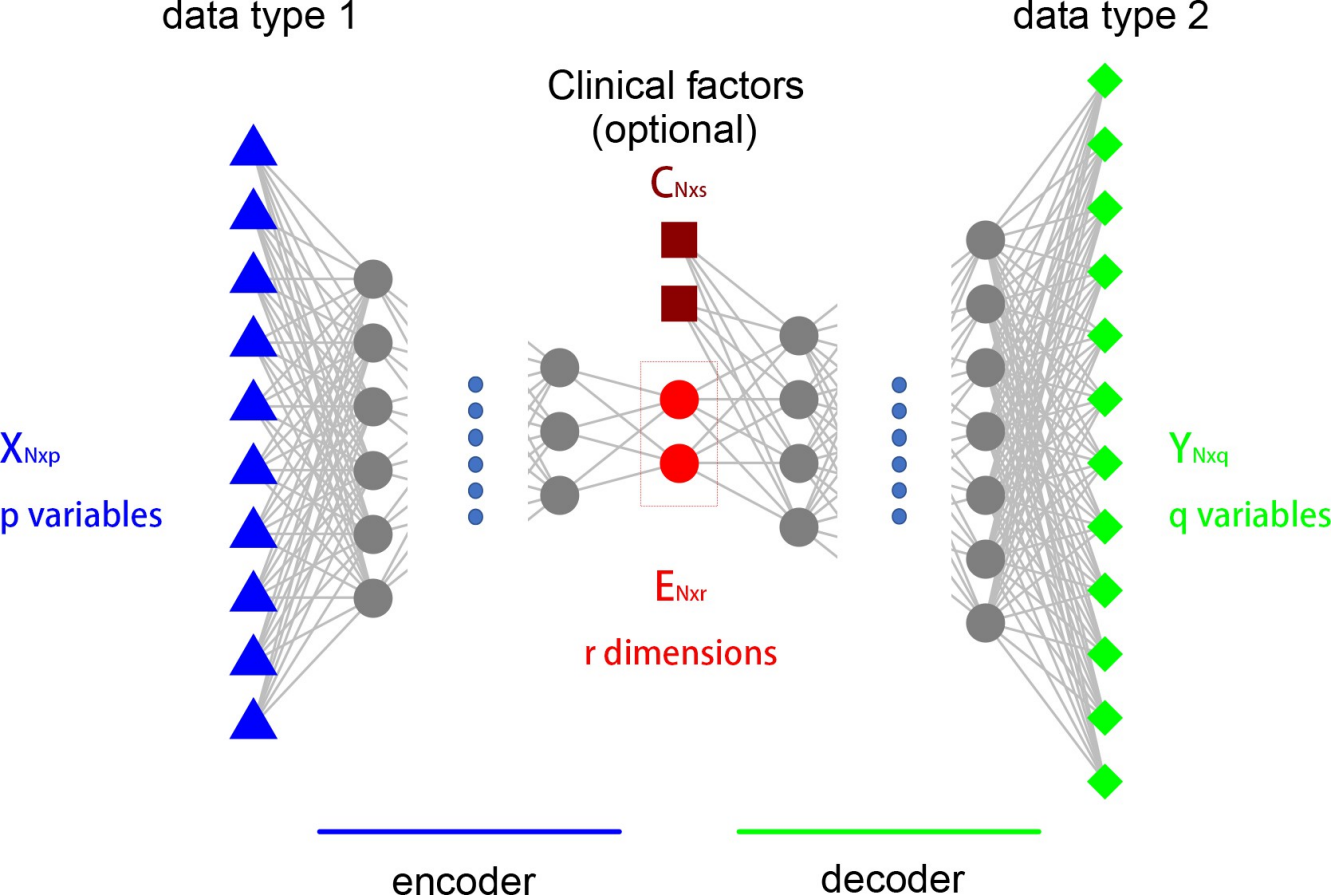
The setup of the model. *X*_*N*×*p*_ is the input data. There are ***p*** variables and ***N*** samples. ***Y***_***N*×*q***_ is the output data. There are ***q*** variables and ***N*** samples. ***E***_***N***×***r***_ is the low-dimensional nonlinear data embedding, where ***r*** is small. Clinical confounders such as age, gender, ethnicity, batch *etc*, form the matrix ***C***_***N***×***s***_.

We set up a neural network structure that is similar to autoencoder, as shown in Fig 1. Different from the typical autoencoder, the input layer and reconstruction layer use two different omics data types. The input layer contains ***p*** variables corresponding to the columns of the ***X*** matrix. The output layer contains ***q*** variables corresponding to the columns of the ***Y*** matrix. In addition to the input data ***X***, clinical confounders such as age, gender, ethnicity, batch *etc*, can form another matrix ***C***_***N***×***s***_, Their effects can be adjusted for by inserting the variables in ***C*** as auxiliary variables at the bottleneck layer. This encourages the model to find nonlinear data embedding of ***X*** that contribute to the reconstruction of ***Y***, independent of the clinical confounders.

In some sense this is a prediction structure with very high dimensional outcome. Such a prediction task is unrealistic, and our goal is not prediction. With a very narrow bottleneck layer in the middle, we essentially seek a nonlinear dimension reduction of the input data ***X***, which best preserves the information pertaining to the output data ***Y***, while adjusting for confounding factors in matrix ***C***. Following traditional statistical terminology in dimension reduction, we call the columns of the embedded data matrix ***E*** “components” in this manuscript.

### 2.2. Implementation

The program was implemented in R using the Keras neural networks API [24], to facilitate users of R to conduct the analysis. The implementation requires both R and the TensorFlow backend. With regard to the sizes of the layers of the network, the method allows three different ways for the user to specify. (1) The user can directly specify the sizes of all the individual layers; (2) the user can input a shrinkage factor, such that the size of each layer in the encoder is the product of the size of the previous layer and the shrinkage factor, and the size of each decoder layer is the product of the next layer and the shrinkage factor; (3) the user can input the desired number of input/out layers, and the shrinkage factor is calculated based on the number of layers. Dropout rates can be specified to be uniform across all layers, or in a layer-by-layer manner.

Given the number of layers and dropout rates, the data is split into training and testing sets. The prediction error rate on the testing set is used to select the number of training epochs. Once the number of epochs is determined, the full dataset is used to fit the model again.

### 2.3. Estimating feature importance

To find which feature from the input matrix ***X*** is more influential, we use a permutation scheme. We fix the parameters in the trained model. In each iteration, one variable in the ***X*** matrix is permuted, and new embedding is calculated based on the existing parameters. We then compare the new embedded data with the embedded data from the unpermuted data. The amount of location shift, measured by the sum of squared distances across all the embedded data points, is taken as the importance of the permuted variable. Similarly, we estimate the pairwise influence, *i*.*e*. the influence of one variable in the ***X*** matrix on one variable in the ***Y*** matrix in the same permutation, by recording the amount of change of each ***Y*** variable, when the ***X*** variables are permuted.

### 2.4. Selecting important input variables and input-output variable pairs using a model-based approach

The importance scores of the input variables are all positive. Based on our empirical observations across multiple datasets, the score distribution of the irrelevant input variables can be modeled very well by the gamma distribution. Thus we adopt a model-based approach that follows the general idea of local false discovery rate (fdr) [25]. In this approach, we consider the observed importance scores to follow a mixture distribution with two components–the null (unimportant variables) distribution that is a gamma distribution with unknown parameters, and the non-null (important variables) distribution with unknown parametric form. We use the following procedure to estimate the fdr of each input variable:

1. Use kernel density estimator to estimate the nonparametric distribution of the importance scores. Find the mode of the distribution.
2. Select the lower portion of the importance scores that are below a certain percentile *π*_0_, and assume most of these selected importance scores are from the null distribution. This is because the null importance scores tend to be low. Using the scores below *π*_0_, estimate the shape and scale parameters of the Gamma distribution.
3. Calculate the sum of squared difference below the mode, between the Gamma density obtained in step (2) scaled by *π*_0_, and the kernel density obtained in step (1). This is the indicator how well the Gamma density fits the null component of the distribution.
4. Vary *π*_0_ from 0.6 to 0.99, with a step size of 0.01. Find the *π*_0_ that yields the smallest difference in step (3).
5. Using the *π*_0_ found in step (4), and the corresponding Gamma density, denoted *f*_0_(), and the kernel density found in step (1), denoted *f*(), find the local fdr value at any given location by fdr(z)=π0f0(z)f(z).
6. Find the smallest *z* value such that *fdr(z)* is below a pre-determined threshold. Any importance score higher than this value is considered significant.

### 2.5. Tuning hyperparameters

In this study, we used the multivariate skewness and kurtosis of the embedded data to select the number of layers and dropout rates. At each hyperparameter setting, the data embedding (matrix ***E***) is computed, and the average absolute pairwise correlation between the columns of the ***E*** matrix is calculated. Among the settings for which the average correlation is below a threshold (indicating the embedded dimensions are not duplicating information), the Mardia’s multivariate skewness and kurtosis coefficients are calculated for the embedded data [26]. We rank each setting by the skewness and kurtosis of the embedded data, and then select the setting that yield the highest average rank of skewness and kurtosis. This process selects parameter settings that yield embedding that is not highly correlated, as well as with a distribution far from multivariate normal. This is because a random projection of the data into lower dimensions tend to yield multivariate normal distribution. The criterion we use here is similar to that of projection pursuit [27].

### 2.6. Simulation study

We use the following procedure to generate simulated data:

1. Generate the ***X*** matrix with *n*_*x*_ variables and *N* samples using multivariate normal distribution. The mean vector is ***0***. The diagonal elements of the variance-covariance matrix *Σ* is 1, and all off-diagonal elements take value *ρ*, which is a value between 0 and 1. Inverse-normal transform *X*, and subtract 0.5, such that the values in the *X* matrix are between -0.5 and 0.5.
2. Select the first *k* variables in the ***X*** matrix. Generate three linear combinations of the *k* variables $z_{m}=\sum_{j=1}^{k}\beta_{mj}x_{j},\;m=1,2,3$, where the *β*′*s* are randomly sampled from a uniform distribution on [−2, −1]∪[1,2].
3. Generate the first *m*×*k* variables in the *Y* matrix, by first generating a linear combination of the *z* variables, *r*_*j*_ = *α*_*j*1_*z*_1_+*α*_*j*2_*z*_2_+*α*_*j*3_*z*_3_, *j* = 1,…,*mk*, where the *α*′*s* are randomly sampled from a uniform distribution on [−2, −1]∪[1,2]. Then the *r* variables are re-scaled to facilitate nonlinear transformation, by subtracting the mean and dividing by 3 times the standard deviation. This transformation ensures most of the *r* values are between -1 and 1. We then take *y*_*j*_ = *f*_*j*_(*r*_*j*_), *j* = 1,…,*mk*, where *f*_*j*_() is sampled from five different function: (1): *f(r) = r*, (2): *f*(*r*) = |*r*|, (3): *f*(*r*) = *sin*((5×(*r*+0.5)×*pi*)), (4): *f*(*r*) = (2*r*)^2^, and (5) a step function that takes value 1 when *r* is between the 25^th^ and 75^th^ *percentiles*, and 0 otherwise. The proportion of *y’s* that receive the original *r* values without any transformation is controlled by a hyperparameter. Gaussian random noise is added to each of the *y* variables, such that the noise variance is 1/10 of that of the *y* variable.
4. The remaining *n*_*y*_−*mk* variables in the *Y* matrix are sampled from multivariate normal distribution of mean vector is ***0***, and variance-covariance matrix with diagonal value 1 and off-diagonal value *ρ*. All the variables in the *Y* matrix are then re-scaled to have mean 0 and standard deviation 1.

We then analyze the simulated data using six methods: AIME, CCA, PLS, MOFA2, jSVD, and iCluster2. For simplicity, we fix the number of input layers and output layers of AIME at 3, and the dropout rate at 0.4. For each of the methods, we test two dimensionalities of the embedded data– 3 and *k*, and report from the setting that yields better results. We compare the variable importance ranking generated by each method, *i*.*e*. whether the first *k* variables in the *X* matrix receive higher importance scores, using the area under the curve (AUC) of the precision-recall (PR) curve.

A number of scenarios, as specified by the combinations of *n*_*x*_, *n*_*y*_, *k*, *m*, *N*, *ρ*, and the proportion of *y’s* that are linearly associated with ***X***, *i*.*e*. receiving the original *r* values without any nonlinear transformation. In each scenario, the simulation was repeated 10 times, and the average PR-AUC value was taken.

### 2.7. Datasets and software implementations

Two datasets were used in the manuscript. The first was the Cancer Cell Line Encyclopedia (CCLE) microRNA and gene expression dataset. The CCLE is a collection of ~1000 cancer cell lines, on which multiple omics measurements were made in order to elucidate the mechanisms of different cancers [28]. The data was downloaded from the CCLE website at https://sites.broadinstitute.org/ccle/. The second dataset was the Cancer Genome Atlas (TCGA) breast cancer (BRCA) microRNA and gene expression dataset [29]. The data was downloaded from the GDC data portal https://portal.gdc.cancer.gov/.

AIME implementation is at https://github.com/tianwei-yu/AIME. The installation can be simply done using devtools::install_github("tianwei-yu/AIME"). Some examples are included in the Github description page. Some key parameters include: ‘ncomp’, the dimension of the embedded data; ‘in.layers’ and ‘out.layers’, the number of layers for the encoder and decoder; ‘max.dropout’, the maximum dropout rate; ‘flat.dropout’, whether a single dropout rate is used across all layers, as opposed to outer layers use higher dropout; ‘max.epochs’, the maximum number of epochs in the training; ‘importance.permutations’, how many permutations to conduct to find variable importance; ‘pairwise.importance’, TRUE/FALSE, whether to compute pairwise importance between input and output variables. For the other parameters and details in the default settings, please refer to the Github webpage and the help file.

The method is somewhat sensitive to the setting of some hyperparameters, namely the number of encoder/decoder layers and dropout rate. When the layers are too few, the embedded data tend to be closer to multivariate Gaussian. When the layers are too many, the embedded data tend to fall on combinations of line-segments. To aid hyperparameter tuning, we provide a utility “aime.select()”, which runs through a number of hyperparameter combinations, and generates a PDF file with embeddings at each setting, as well as the skewness/kurtosis measurements at each setting, for the user to conduct an informed hyperparameter selection.

In the simulations and data analyses, we compared with the methods CCA, PLS, iCluster2, jSVD and MOFA2. For CCA and PLS, we used the implementation in the Bioconductor package mixOmics [30]. For MOFA2, we used the implementation downloaded from Bioconductor [8]. For iCluster2, we used the CRAN package iCluster [6,31]. For jSVD, we used the CRAN package multiway [32].

## 3. Results and Discussion

### 3.1 Simulation results

**Fig 2:**
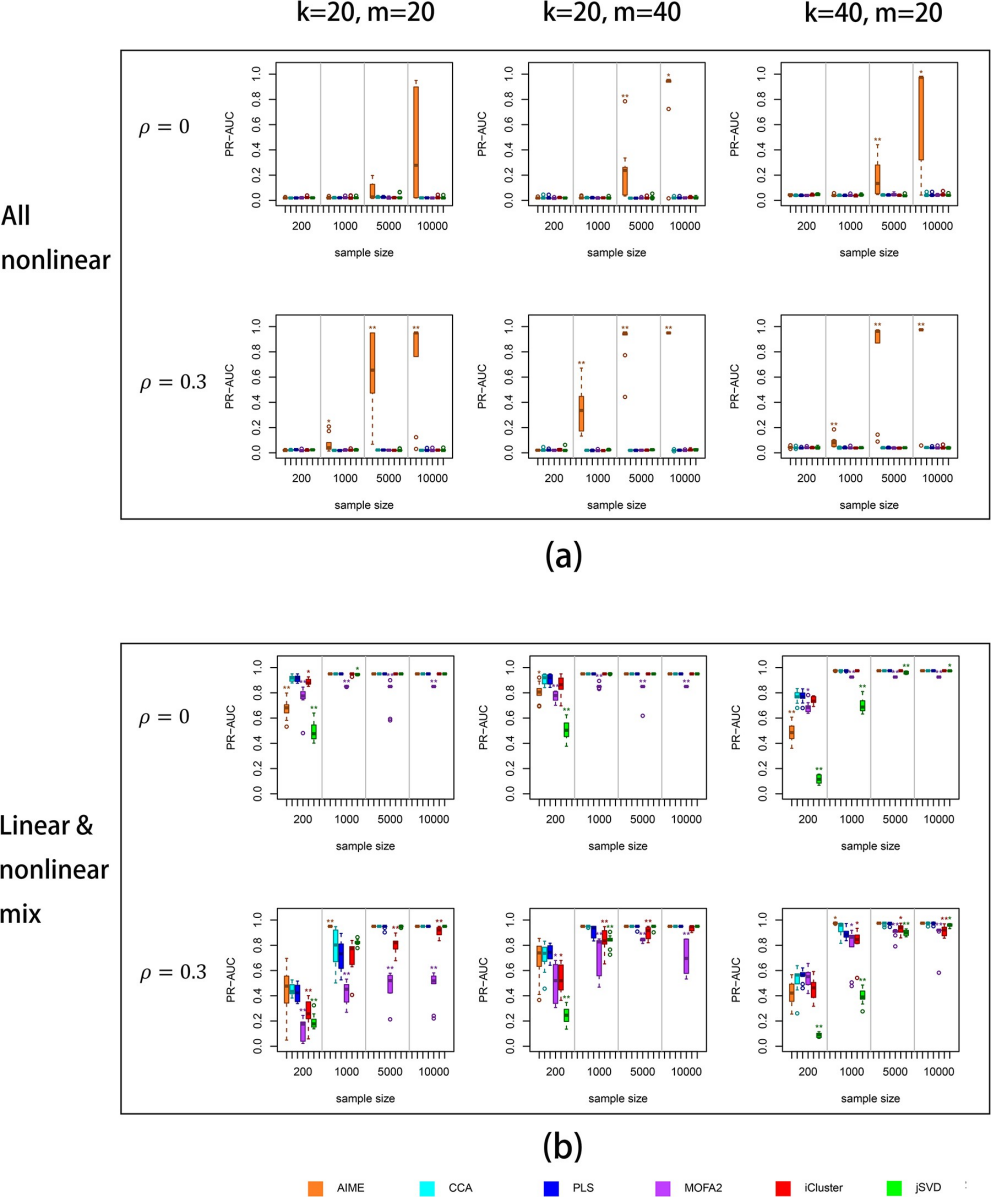
Simulation results. PR-AUC was used to assess each method’s success in selecting the true contributing variables. (a) All relations are nonlinear; (b) The relations are mixed between nonlinear and linear. X-axis: sample size; Y-axis: PR-AUC values. For full simulation results, please see S1 Fig. The significance levels by Wilcoxon test against the CCA results were labeled on the plots: p≤0.01 (*); p≤0.001(**).

The simulation results are shown in Fig 2. Because most of the simulated ***X*** variables do not contribute to the relation with ***Y***, we used the area under the precision-recall curve (PR-AUC) to assess the methods’ capability to separate the truly contributing ***X*** variables from the rest. We present part of the simulation results in Fig 2, and the full simulation results are in S1 Fig. For AIME and jSVD, the embedded dimension *k* yielded better results, while for iCluster2, MOFA2, PLS and CCA, the embedded dimension of 3 yielded better results.

When the relation between ***X*** and ***Y*** were purely non-linear (Fig 2A), among all the six methods, only AIME could extract the contributing ***X*** variables. However, it required the sample size to be relatively large. For example, when 20 ***X*** variables contributed to the relation between ***X*** and ***Y***, and 400 ***Y*** variables (out of 1000) were impacted, AIME achieved reasonably good PR-AUC when the sample size was 5,000 or higher and there is some moderate correlation (*ρ* = 0.3) between the ***X*** variables. When higher number of ***Y*** variables were associated with the ***X*** variables (800 out of 1000; Fig 2A, middle and right column), the power to select the contributing ***X*** variables became higher. Interestingly, we observed that the power was higher when there was a moderate level of correlations (0.3) among all the ***X*** variables (Fig 2A, second row), as compared with the scenarios of no correlation (Fig 2A, first row). Overall, due to the intrinsic difficulty in capturing nonlinear relations, usually higher sample size (≥1000) was required to achieve moderate or higher PR-AUC levels.

We next examined the situation where the relation between ***X*** and ***Y*** were mixed (Fig 2B). When there was no correlation between the ***X*** variables (Fig 2B, first row), all six methods performed similarly, with PLS, CCA and iCluster2 leading the performance when the sample size was small. AIME, PLS, CCA and iCluster2 performed similarly when the sample size was moderate (1000) or higher. When some pervasive correlations exist between the ***X*** variables (*ρ* = 0.3), AIME achieved similar level of performance as PLS and CCA at very low sample size of 200, indicating the capability of neural networks even in the situation of *N<<p*, i.e. sample size much smaller than variable count. When the sample size was moderate (1000) or higher, AIME actually led the pack in performance.

When all the relations between ***X*** and ***Y*** were linear (S1 Fig, right column), the relative performance of the methods were similar to that of the mixed situation (Fig 2B). Again we saw AIME was comparable to PLS, CCA, iCluster2 and MOFA2 even at very small sample size, and rose to an overall better performance when the sample size was moderate (1000) or higher, especially in cases where correlations exist between the X variables (S1 Fig, right column). Overall, AIME was the only method that could pick up nonlinear relations, and it was a strong competitor when the signal was linear or mixed.

### 3.2 Cancer Cell Line Encyclopedia (CCLE) microRNA and gene expression dataset

In this study, we analyzed the sequencing-based microRNA and gene expression data of CCLE. After mapping cell lines between the two omics data types, the datasets contained 942 cell lines. The data matrices were transformed by *log*_*10*_*(x+1)* transformation. We filtered the miRNA data using the criterion of Coefficient of Variation (CV) >0.1, which yielded 700 miRNAs. We filtered the gene expression data using the criteria of percent of zeros < 25%, and CV > 0.5, which yielded 14997 genes.

After selection of hyperparameters, we used the setting of 3 input layers, 4 output layers, and dropout rate of 0.3. We also ran CCA, PLS, iCluster2, SNF, jSVD and MOFA2 on the dataset. The PLS results were qualitatively similar to the CCA results. Hence we present the results from AIME, CCA, iCluster2, jSVD, SNF and MOFA2 here. As SNF doesn’t directly produce data embedding, we resorted to applying PCA on the overall status matrix derived by SNF. For both CCA, iCluster2, jSVD, SNF and MOFA2, we tried both the original data after log transformation, as well as standardizing the data by removing mean and dividing by standard deviation for each variable. The embedding results were similar. Here we present results from the standardized data.

**Fig 3:**
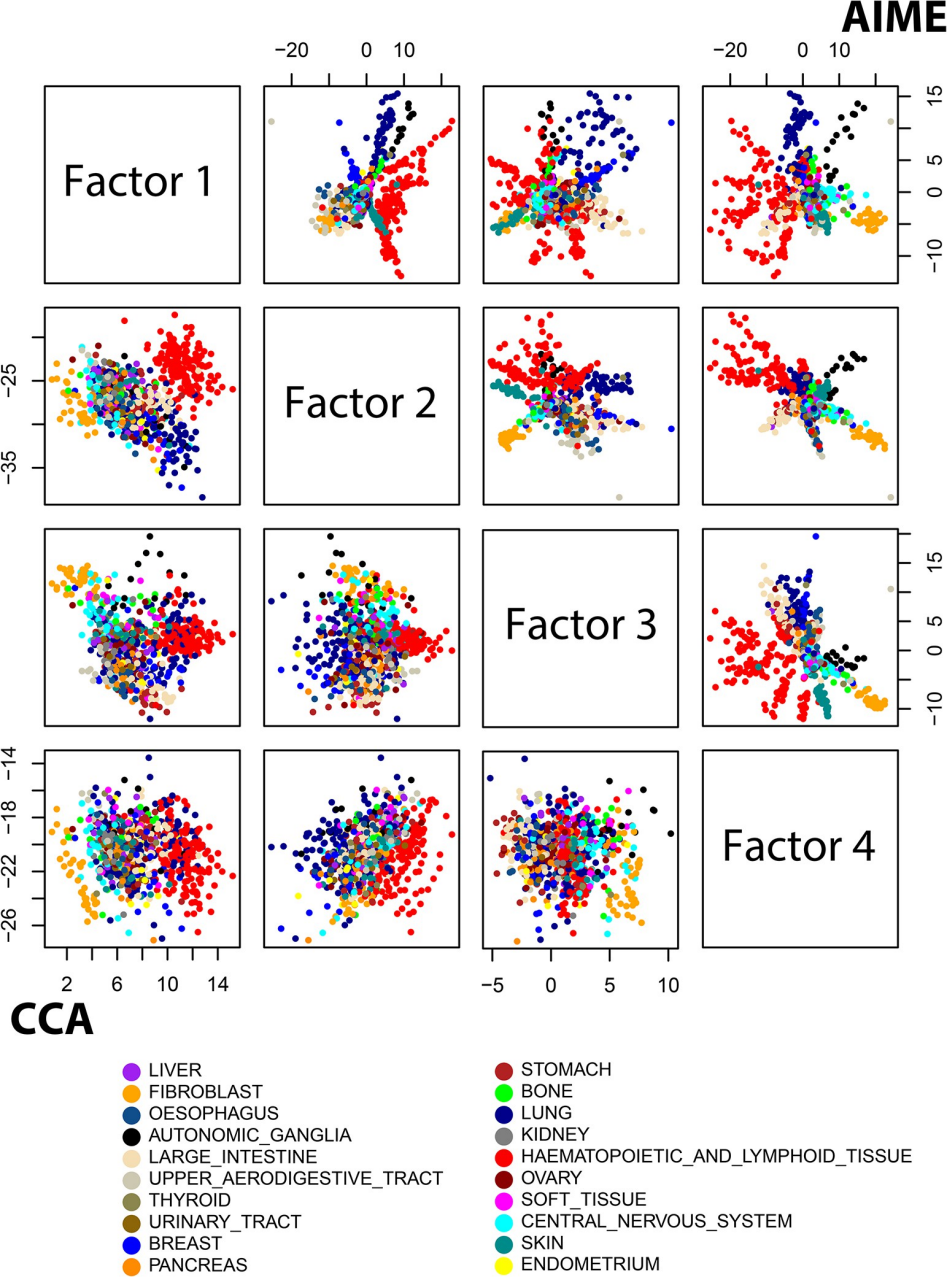
Comparing AIME and CCA results using the CCLE microRNA and gene expression data. The corresponding MOFA2 results are in S2 Fig.

Fig 3 shows the data embedding obtained by AIME and CCA. Each point represents a cell line. AIME (Fig 3, sub-plots in the upper-right triangle) separated several types of cancers from the others, such as haematopoitic and lymphoid cancers (red), autonomic ganglia cancer (black), pancreas cancer (orange), and skin cancer (dark cyan). In addition, some cancer types were further separated into clusters. On the other hand, the data points were more mixed in the CCA embedding (Fig 3, sub-plots in the lower-left triangle), and there was no finer clustering pattern within each color. The behavior of MOFA2, iCluster2, and jSVD were qualitatively similar to that of CCA, with iCluster2 and jSVD showing more cancer type separation (S2, S3 and S4 Figs). SNF yielded a distinctively different pattern (S5 Fig), with some classes better separated.

**Fig 4:**
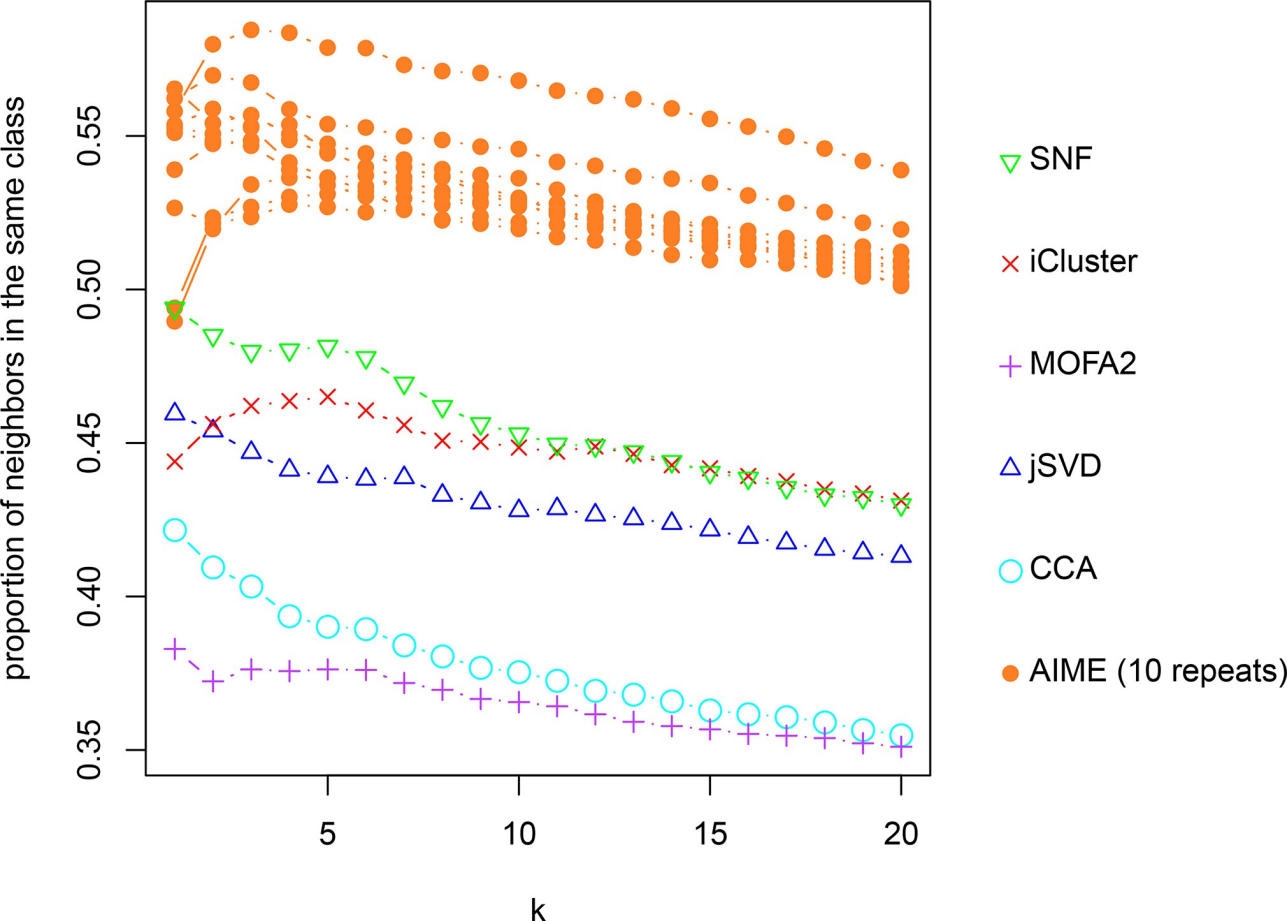
Proportion of nearest neighbors of each data point to be from the same cancer class in the embedded data. The k-nearest neighbors (k = 1 to 20) were considered. For AIME, results from 10 repeats were presented.

As pairwise plots may be misleading in terms of class separation, we further analyzed the embedded data. We examined the k-nearest neighbors (k = 1~20) of each data point, and calculated the average proportion of the neighbors being from the same cancer type. Given that AIME model fitting is stochastic, we repeated the process 10 times, and plotted results from the 10 repeats, which agree reasonably well (Fig 4). Overall, AIME attained the highest proportion at all k values, followed by SNF and iCluster2.

**Fig 5:**
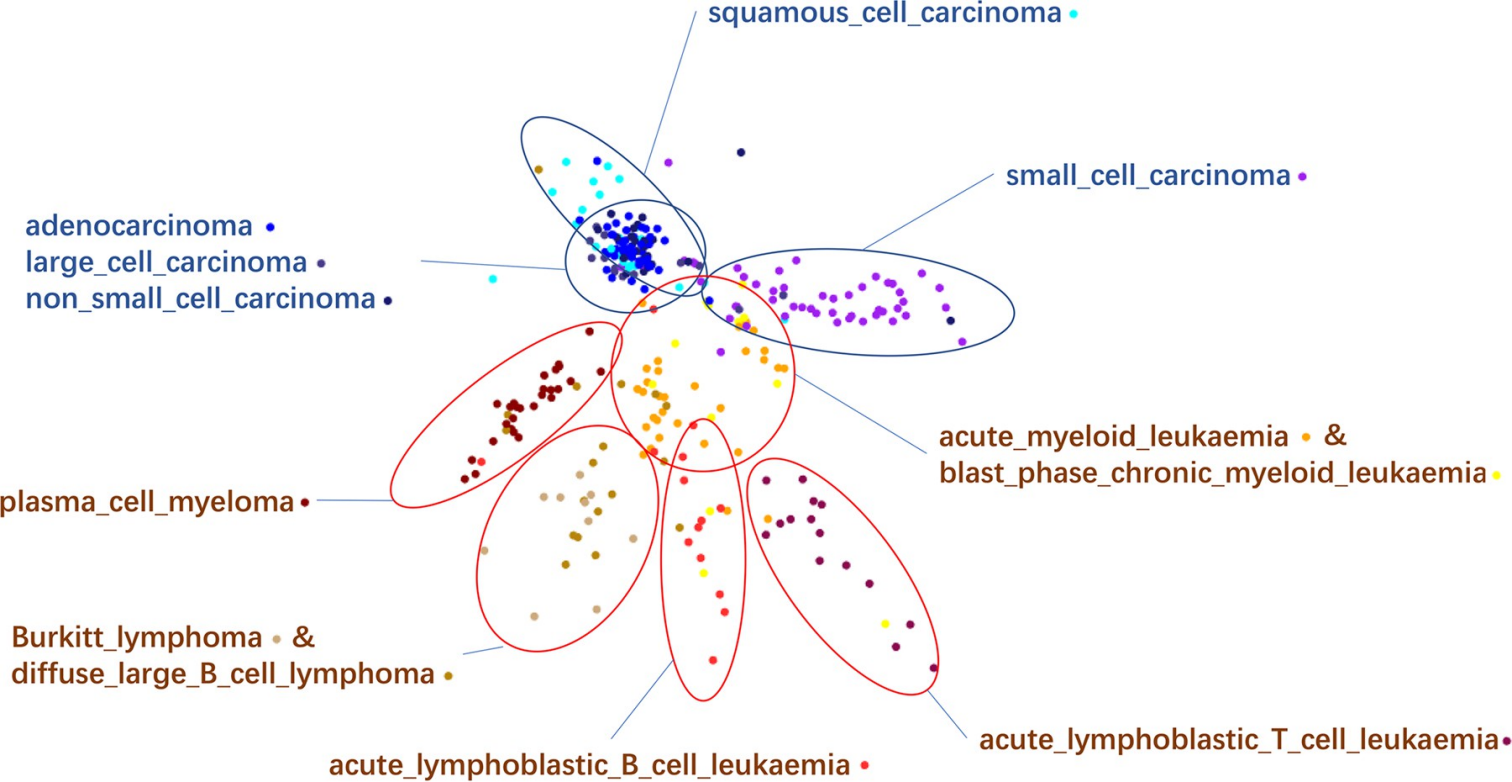
Detailed examination of the two dominant cancer types: “lung” and “haematopoietic and lymphoid tissue”. The points are colored using the cancer subtypes. The dominant subtypes of each region are labeled on the plot. Blue circle and text: lung cancer subtypes; brown circle and text: subtypes of cancer of haematopoietic and lymphoid tissue origin. When two subtypes are inseparable, their subtypes labels are written together.

As we noticed finer cluster structure within some cancer types in the AIME results, we further examined the AIME embedding by taking the two largest cancer types, lung cancer and haematopoietic and lymphoid cancer. We colored the data points by cancer sub-types, and manually rotated the view within the first 3 dimensions of the AIME-embedded data (Fig 5). We found that the AIME embedding separated some cancer subtypes reasonably well. Among the lung cancer sub-types (blue text labels in Fig 5), small cell carcinoma was clearly separated from the rest, and squamous cell carcinoma was partially separated. The other three subtypes: adenocarcinoma, large cell carcinoma and non-small cell carcinoma were mixed together, indicating their molecular similarity.

There are many subtypes of haematopoietic and lymphoid cancer. Some were clearly separable from the rest, including acute lymphoblastic T cell leukemia, acute lymphoblastic B cell leukemia, and plasma cell myeloma (brown text labels in Fig 5). Some subtype pairs were mixed together, but separated from other subtypes, such as Berkitt lymphoma and diffuse large B cell lymphoma. Acute myeloid leukemia and blast phase chronic myeloid leukemia were also mixed together, but partially separable from the rest.

Overall, AIME was able to distinguish cancer types and sub-types much clearer in its embedding, as compared to CCA, iCluster2, jSVD, SNF and MOFA2. We then examined the most influential miRNAs found by AIME. We ran the same parameter setting 10 times, and averaged the variable importance scores. The agreement between the 10 runs were very good, the average pairwise correlation between the importance scores being 0.84. We set the fdr threshold at 0.001, which yielded 35 miRNAs (S6 Fig).

For each of these 35 miRNAs, we used the same gamma distribution-based fdr procedure to select the genes most influenced by it. Again the miRNA-gene influence score was aggregated from 10 runs at the same hyperparameter setting. The 10 runs had very good agreement, with an average correlation of 0.73 between the miRNA-gene influence scores. S6 Fig shows an example local fdr fitting result. We note that some miRNAs showed a strong impact on a specific set of genes, while some others have a more non-specific impact, indicated by their high overall impact score, yet very few genes were selected for the miRNA using the local fdr procedure, as the local fdr procedure only select genes that “stand out” from the background. As a compromise, when the local fdr procedure selected less than 10 genes for a specific miRNA, we allowed its top 10 genes to enter the list.

The above procedure yielded a total of 2579 miRNA-gene relations at the miRNA-gene fdr level of 10^−4^. Among them, 358 were validated by the multiMir package, which queries multiple sources for experimentally and computationally validated miRNA targets [33]. The proportion of the relations validated (13.9%) is 2.06-fold of the background (6.73%) among the 35 miRNAs and all the genes under study, indicating an informative selection of miRNA-gene pairs.

As there were two fdr thresholds involved in the selection, to ensure the robustness of the results, we varied the fdr threshold for miRNA importance, as well as the fdr threshold miRNA-gene pairs, and calculated the ratio between the validated proportion of selected miRNA-gene pairs v.s. the background validation rate of the selected miRNAs. As shown in S7 Fig, the ratio tended to be higher when both fdr thresholds were more stringent.

**Fig 6:**
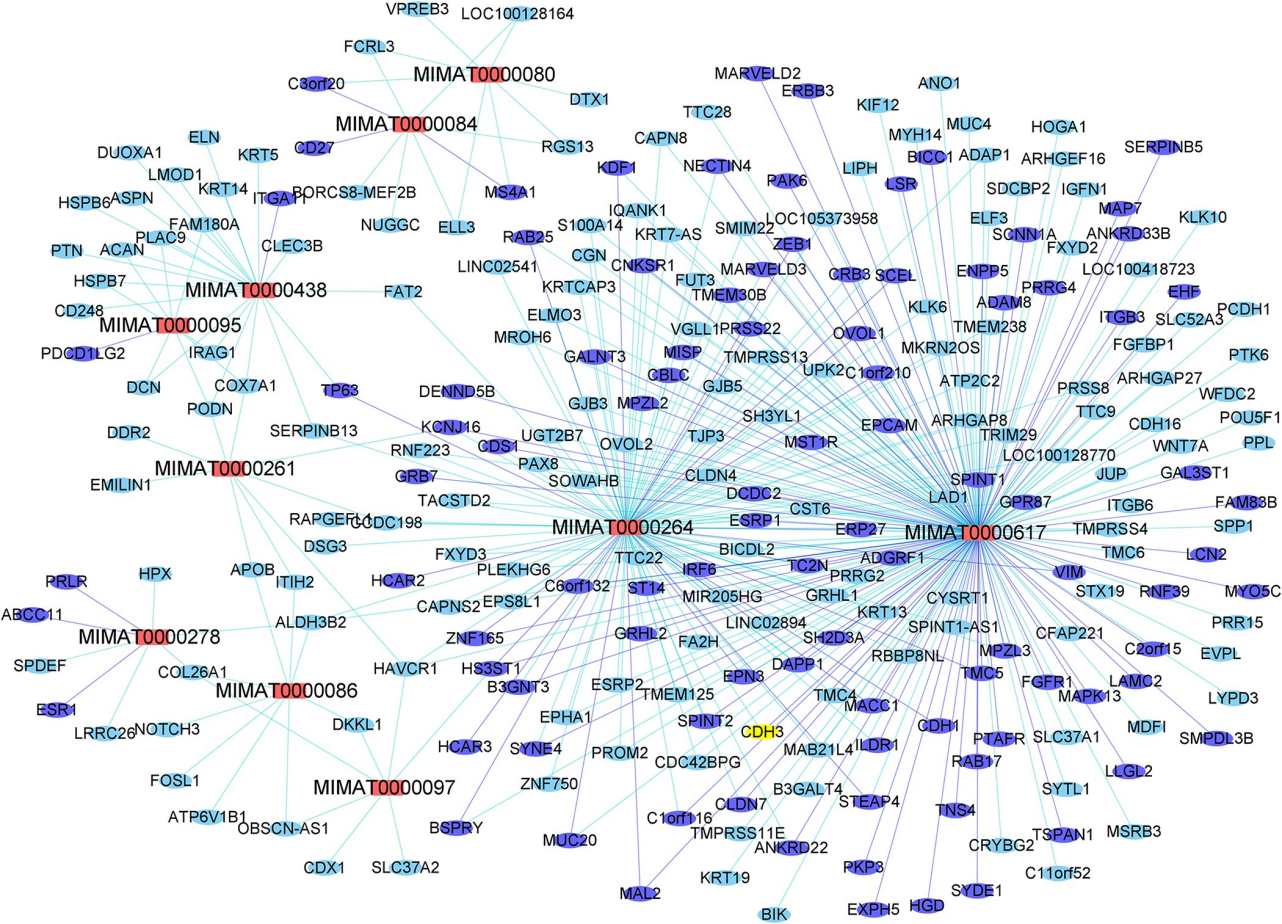
Top 10 microRNAs (fdr≤10^−7^) and their associated genes (fdr≤10^−5^) in the CCLE data. Red nodes: microRNAs; blue nodes/edges: validated by multiMir.

For illustration purposes, we further narrowed down to the top 10 miRNAs by using an fdr threshold of 10^−7^. For these 10 miRNAs, a total of 437 miRNA-gene pairs were identified at miRNA-gene fdr of 10^−5^, among which 110 (25.2%) were validated by multiMir, representing a 3.28-fold increase over the baseline. The miRNA-gene graph is shown in Fig 6, with validated miRNA-gene relations colored in blue. Among the two miRNAs with highest number of connections, MIMAT0000264 (has-mir-206) is a known suppressor of breast cancer metastasis [34]; MIMAT0000617 (hsa-miR-200c) is a known regulator of pancreatic cancer invasion and proliferation [35].

**Fig 7:**
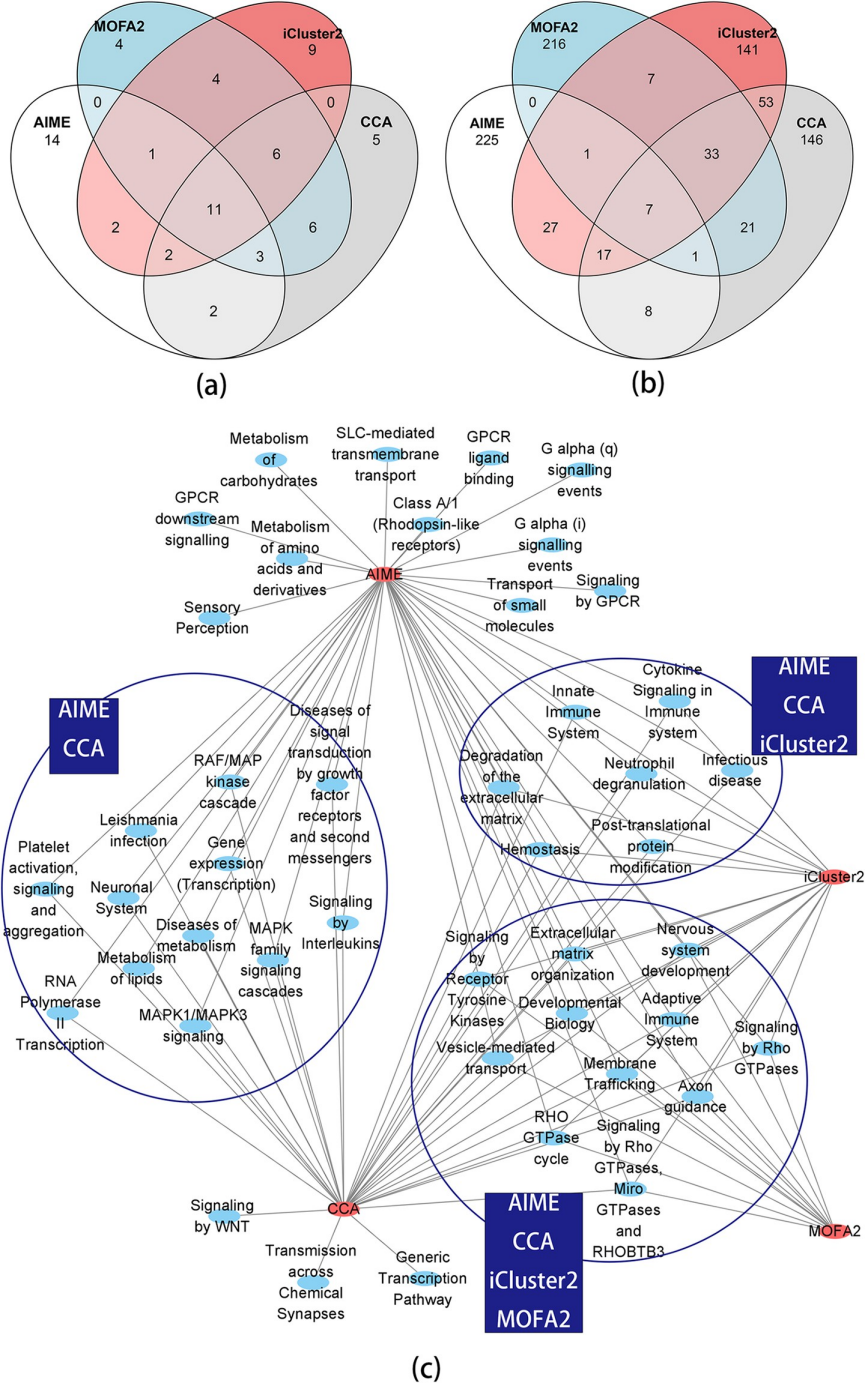
Venn Diagram of the top microRNAs, top genes, and functional analysis of the top genes. Notice the functional analysis results of the microRNAs are in Table 1. (a) Venn diagram of the top 35 microRNAs selected by AIME (fdr≤0.01), and the top 35 microRNAs from CCA, iCluster2 and MOFA2. (b) Venn diagram of the top 286 genes selected by AIME (fdr≤0.05), and the top 286 genes from CCA, iCluster2 and MOFA2. (c) The overrepresented pathways (FDR≤0.05) with 100~500 genes.

For a further functional comparison between AIME, CCA, iCluster2 and MOFA2 results, we used the top 35 miRNAs selected by AIME. Given the other methods do not generate p-value or fdr for miRNAs, we selected the top 35 miRNAs using CCA, iCluster2, and MOFA2 respectively, by ranking the sum of squared loading of the miRNAs. There were reasonably good overlap between the lists of miRNAs selected by the methods (Fig 7A). 11 miRNAs were selected by all 4 methods. CCA and MOFA2, both of which are linear methods, showed better agreements by sharing another 12 selected miRNAs. AIME selected 14 miRNAs that didn’t overlap with the other 3 methods.

Using mirPath 3.0 [36], we compared the functional annotations of the 14 miRNAs selected by AIME alone, versus the 11 miRNAs that were shared by all four methods (Table 1). P-values by mirPath 3.0 were adjusted to FDR using the Benjamini-Hochberg method. Among the top 10 pathways associated with the 14 miRNAs selected by AIME, 3 were insignificant (FDR>0.2) when analyzing the 11 miRNAs selected by all methods. They include a signaling pathway closely related to cancer—PI3K-Akt signaling pathway, as well as a pathway that is critical in cancer cell microenvironment—extracellular matrix (ECM) interactions. The results indicated that AIME was able to identify some important aspects of the data that complement other existing methods.

**Table 1:** Top 10 pathways of selected miRNAs from the CCLE data using mirPath 3.0^#^.

KEGG pathway	FDR	#miRNAs
**AIME only** (14 miRs; total 35 pathways with FDR≤0.1)		
**ECM-receptor interaction** *	3.70E-13	13
**Fatty acid biosynthesis** *	6.78E-10	5
Signaling pathways regulating pluripotency of stem cells	8.42E-10	13
Hippo signaling pathway	2.88E-06	12
Pathways in cancer	3.51E-06	14
Proteoglycans in cancer	9.40E-06	13
Glioma	1.16E-05	12
Focal adhesion	1.68E-05	14
TGF-beta signaling pathway	1.68E-05	12
**PI3K-Akt signaling pathway** *	2.43E-05	14
**All 4 methods** (11 miRs; total 25 pathways with FDR≤0.1)		
Proteoglycans in cancer	5.79E-05	11
MAPK signaling pathway	0.000217	11
ErbB signaling pathway	0.000521	11
Neurotrophin signaling pathway	0.005757	11
Rap1 signaling pathway	0.005757	11
TGF-beta signaling pathway	0.016259	10
Renal cell carcinoma	0.025283	10
Long-term depression	0.025283	10
Thyroid hormone signaling pathway	0.025283	11
Hippo signaling pathway	0.025621	10

* Insignificant at FDR≤0.2 in the “all 4 methods” group

We next examined the top genes selected by the four methods. While AIME selected miRNA-gene pairs, the other three methods do not yield such detailed results. For a fair comparison, for each gene, we aggregated the impact scores from all miRNAs, and selected the top 286 (fdr≤0.05) genes using the gamma distribution-based local fdr procedure. Given the other methods do not generate p-value or fdr for genes, we also selected the top 286 genes by CCA, iCluster2 and MOFA2 respectively, by ranking the sum of squared loading of the genes. iCluster2 and CCA had a moderate level of overlap, while AIME and MOFA2 had low overlap with the other methods (Fig 7B).

We then conducted gene set enrichment analysis using the fast GSEA (fgsea) package [37], which uses the ranking information of all genes. We selected reactome pathways that contain 100~500 genes, with FDR≤0.05 (Fig 7C). The majority of the pathways found by any of the four methods were either signal transduction pathways or immune system pathways. There were also a small number of metabolism/transport pathways. AIME yielded the most number of significant pathways, including uniquely identifying G-protein-coupled receptor (GPCR) pathways that play significant roles in cancer. We note that the pathways on the miRNA side were mostly signally pathways, while the pathways on the gene side include more down-stream processes. This agrees with the fact that miRNAs play a more regulatory role, and through signal transduction, they can impact a variety of downstream processes.

Overall, we found that AIME yielded results that complement existing methods. Given the complexity of the data, each method may only extract part of the information. Combined with the fact that AIME generated embedding that better separated cancer types and sub-types, we believe that AIME detected patterns in the data that can add to what was extracted by existing methods.

### 3.3 TCGA BRCA microRNA and gene expression dataset

We analyzed the TCGA breast cancer microRNA and gene expression datasets [29]. After log-transforming both data matrices, we matched the common subjects in which both data were measured. We then filtered the microRNA data by selecting microRNAs with a coefficient of variation (CV) larger than 0.25, and filtered the gene expression data by selecting genes with <20% zeros, and with a CV larger than 0.2. The resulting matrix dimensions were 451×242 for the microRNA data, and 451×6086 for the gene expression data.

**Fig 8:**
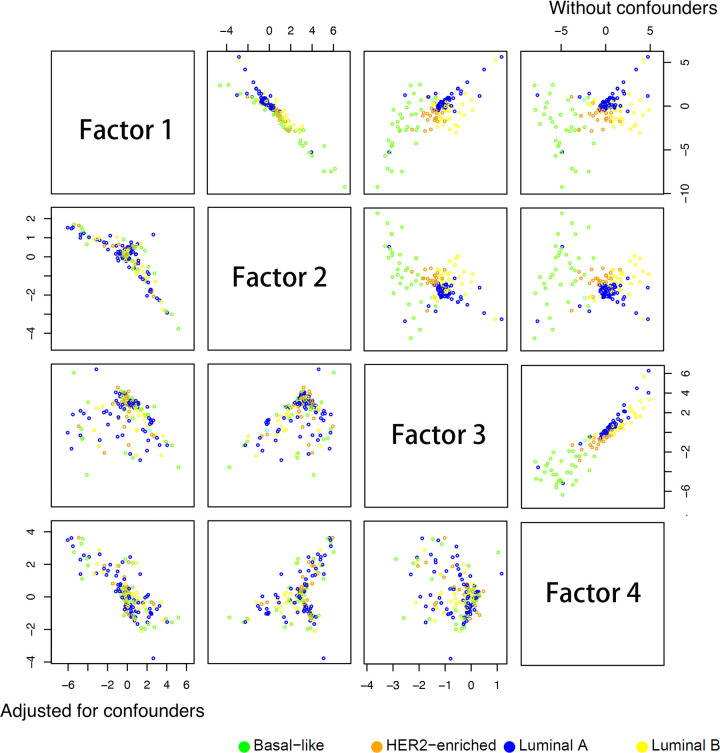
AIME results using TCGA miRNA and gene expression data, with and without adjusting for confounders including age, T1 (tumor size) status, and estrogen receptor (ER) status. Points are colored based on PAM50 (Prosigna Breast Cancer Prognostic Gene Signature Assay) subtypes. Upper-right sub-plots: without adjustment for confounders; lower-left sub-plots: with adjustment for confounders.

We then used the microRNA data as input, and the gene expression data as output. First we ran the analysis without adjusting for any confounder. The method selected 4 layers for the encoder and 5 layers for the decoder, and a dropout rate of 0.2. The resulting embedded data is shown in Fig 8, upper-right triangle. We can see that clearly the embedded data were separated based on the PAM50 (Prosigna Breast Cancer Prognostic Gene Signature Assay) subtypes, which is based on a multi-gene signature for risk stratification [38]. As the ER (estrogen receptor) status is highly correlated with the PAM50 score, the embedded data also separated the subjects based on ER status very well (S8 Fig). Analyzing the dataset using MOFA2 (S9 and S10 Figs), CCA (S11 and S12 Figs), jSVD (S13 and S14 Figs), iCluster2 (S15 and S16 Figs), and SNF (S17 and S18 Figs) yielded similar separations. All methods achieved a separation with the subtype “Luminal A” on one side, and “Basal-like” on the other end. The other two sub-types were in the middle.

**Fig 9:**
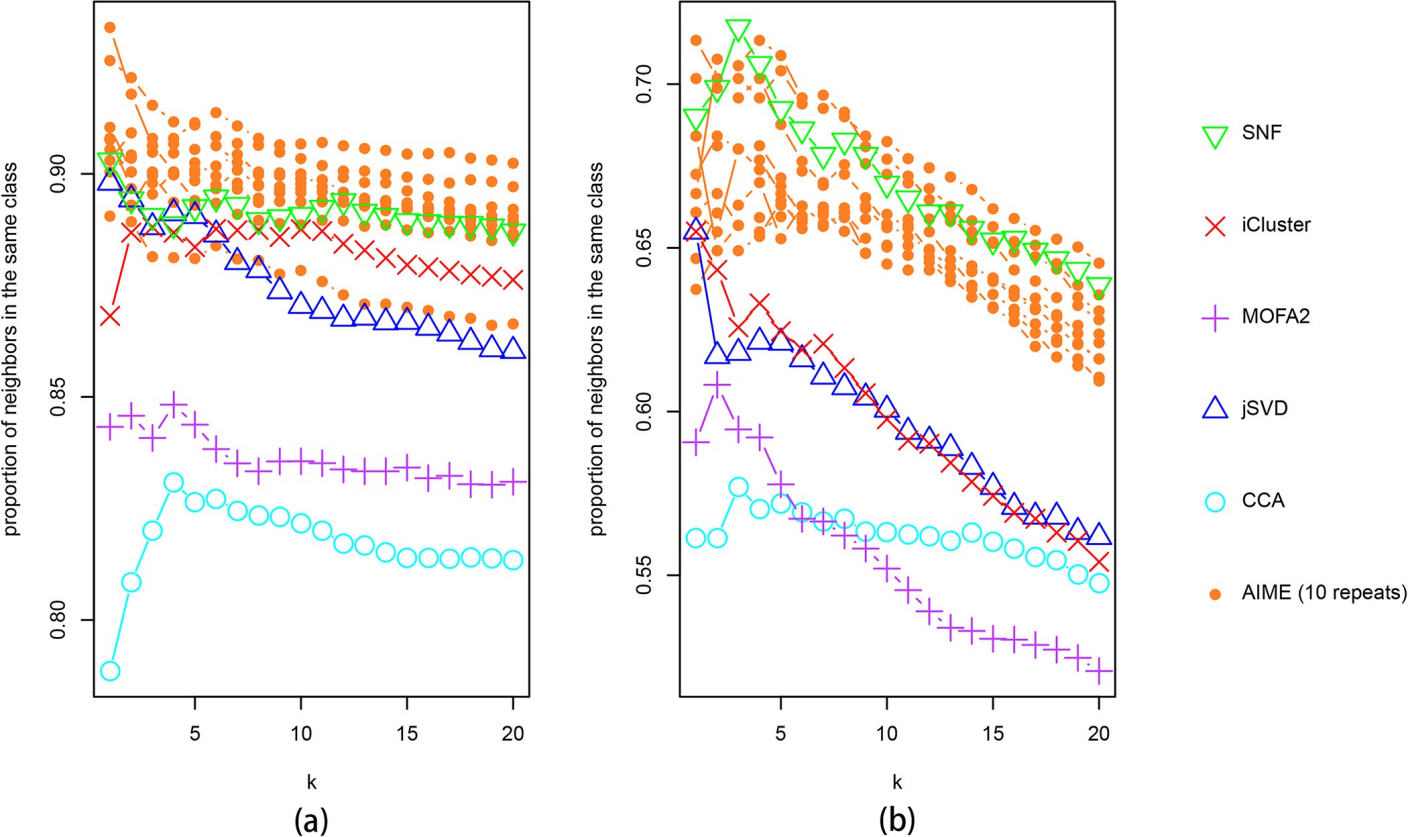
Proportion of nearest neighbors of each data point to be from the same class in the embedded data. The k-nearest neighbots (k = 1 to 20) were considered. (a) Using ER status as class label; (b) using PAM50 group as class label. For AIME, results from 10 repeats were presented.

Again, as pairwise plots may be misleading, we further analyzed the embedded data. We examined the k-nearest neighbors (k = 1~20) of each data point, and calculated the proportions of the neighbors being from the same cancer type. We repeated the AIME analysis 10 times, and plotted results from the 10 repeats, which agree reasonably well (Fig 9). Overall, AIME and SNF attained better results, with AIME separating the ER groups better, and SNF separating the PAM50 groups better.

It is well-known that ER status is a major factor in breast cancer, and this cancer subtype could dominate miRNA and gene expression patterns. It is unclear whether the embedded point patterns were caused by the dominating ER factor, and whether the impact on expression by T1 status (tumor size) overlaps that of ER status. Among all the methods tested, only AIME can answer these questions, be adding ER status and/or T1 status as confounders in the model.

First, we adjusted for age+T1 and age+ER separately. Adjusting for age+T1 wasn’t able to remove the point separation between PAM50 subtypes (S19 Fig) or ER status (S20 Fig), indicating if the T1 status has some impact on the miRNA-gene relations, the signal is unrelated to that of ER status. As expected, when adjusting for age+ER status, the separation by PAM50 or ER disappeared (S21 and S22 Figs).

To maximally remove the impact of the known factors, we then adjusted for age, T1 (tumor size) status, and ER status in the analysis. The purpose was to find any miRNA-gene relations that were independent from age, T1, and ER status. The resulting embedded data clearly lost the relation with PAM50 subtypes, with all four PAM50 subtypes mixed together (Fig 8, subplots in the lower-left triangle). Similar effect was observed with ER status (S8 Fig, subplots in the lower-left triangle).

In the adjusted analysis, the method selected 5 layers for the encoder, 4 layers for the decoder, and a dropout rate of 0.2. We repeated the analysis 10 times at this setting, and averaged the importance scores. At the fdr level of 0.1, sixteen miRNAs were significant based on the gamma distribution-based fdr analysis. The same procedure as in the previous section was used to select miRNA-gene pairs. At the miRNA-gene fdr level of 0.01, a total of 2646 miRNA-gene relations were identified, among which 334 were validated by the multiMir package [33]. The proportion of the relations validated (12.6%) was 1.51-fold of the background (8.3%) among the 16 miRNAs and all the genes under study, indicating an informative selection of miRNA-gene pairs. The fold change was not as substantial as in the CCLE data. However we note that due to the data being from the same cancer, and the adjustment for ER and T1 status, the signal was more subtle in the current analysis. To ensure the robustness of the results, we varied the fdr threshold for miRNA importance, as well as the fdr threshold miRNA-gene pairs, and calculated the ratio between the validated proportion of selected miRNA-gene pairs v.s. the background validation rate of the selected miRNAs. As shown in S23 Fig, the ratio tended to be higher when both fdr thresholds were more stringent.

**Fig 10:**
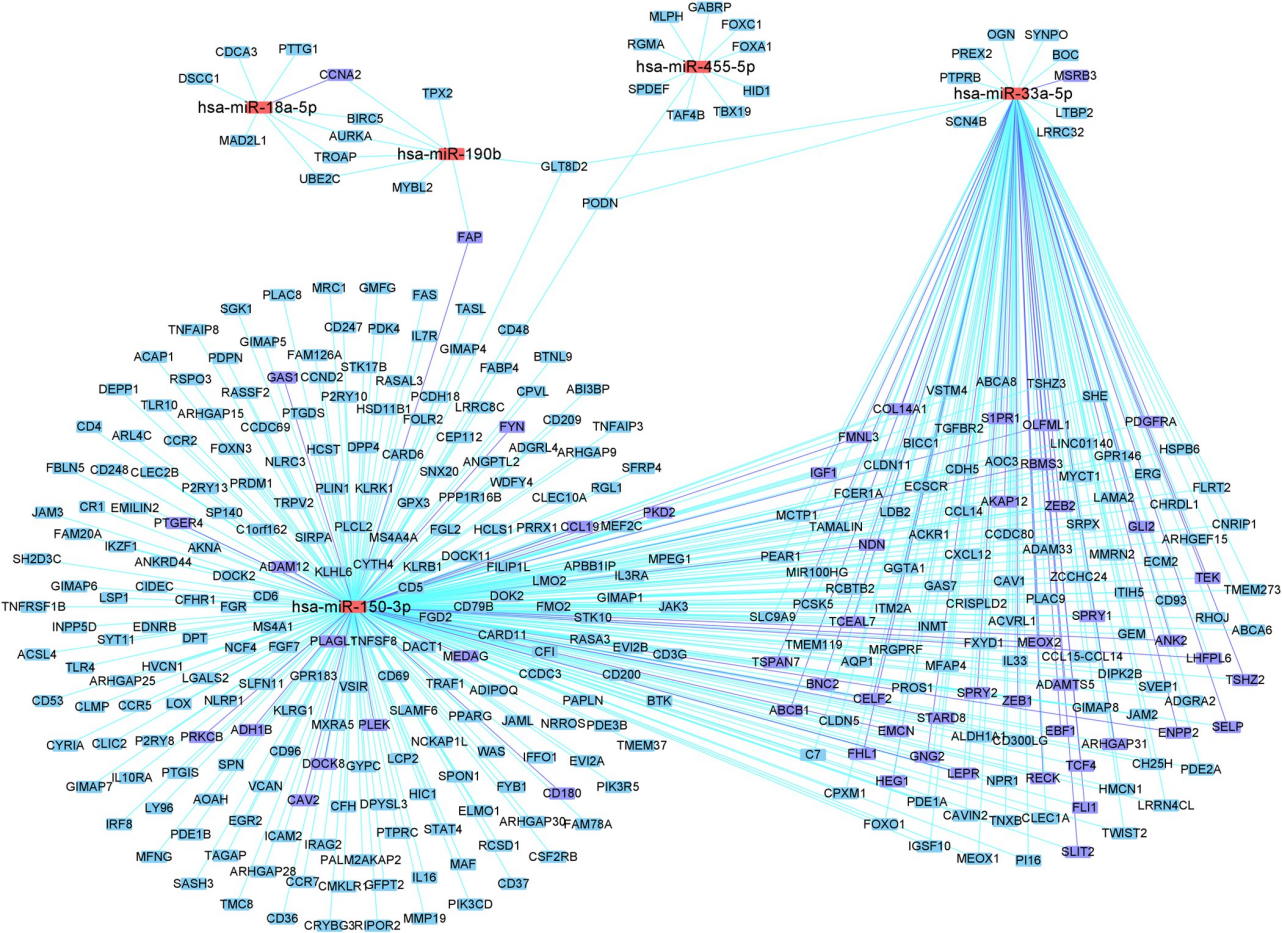
The top 5 microRNAs (fdr≤0.01) and their major associated genes (fdr≤0.001), after adjusting for age, T1 (tumor size) status, and estrogen receptor (ER) status. Red nodes: microRNAs; blue nodes/edges: validated by multiMir.

For illustration purposes, we further narrowed down to the top 5 miRNAs by using an fdr threshold of 0.01. For these 5 miRNAs, a total of 507 miRNA-gene pairs were identified at the miRNA-gene fdr of 0.001, among which 61 (12.0%) were validated by multiMir, representing a 1.73-fold increase over the baseline (6.96%). The miRNA-gene graph is shown in Fig 10. Both of the two miRNAs with large number of connections, mir-33a and mir-150 are known to be associated with many tumors.

**Fig 11:**
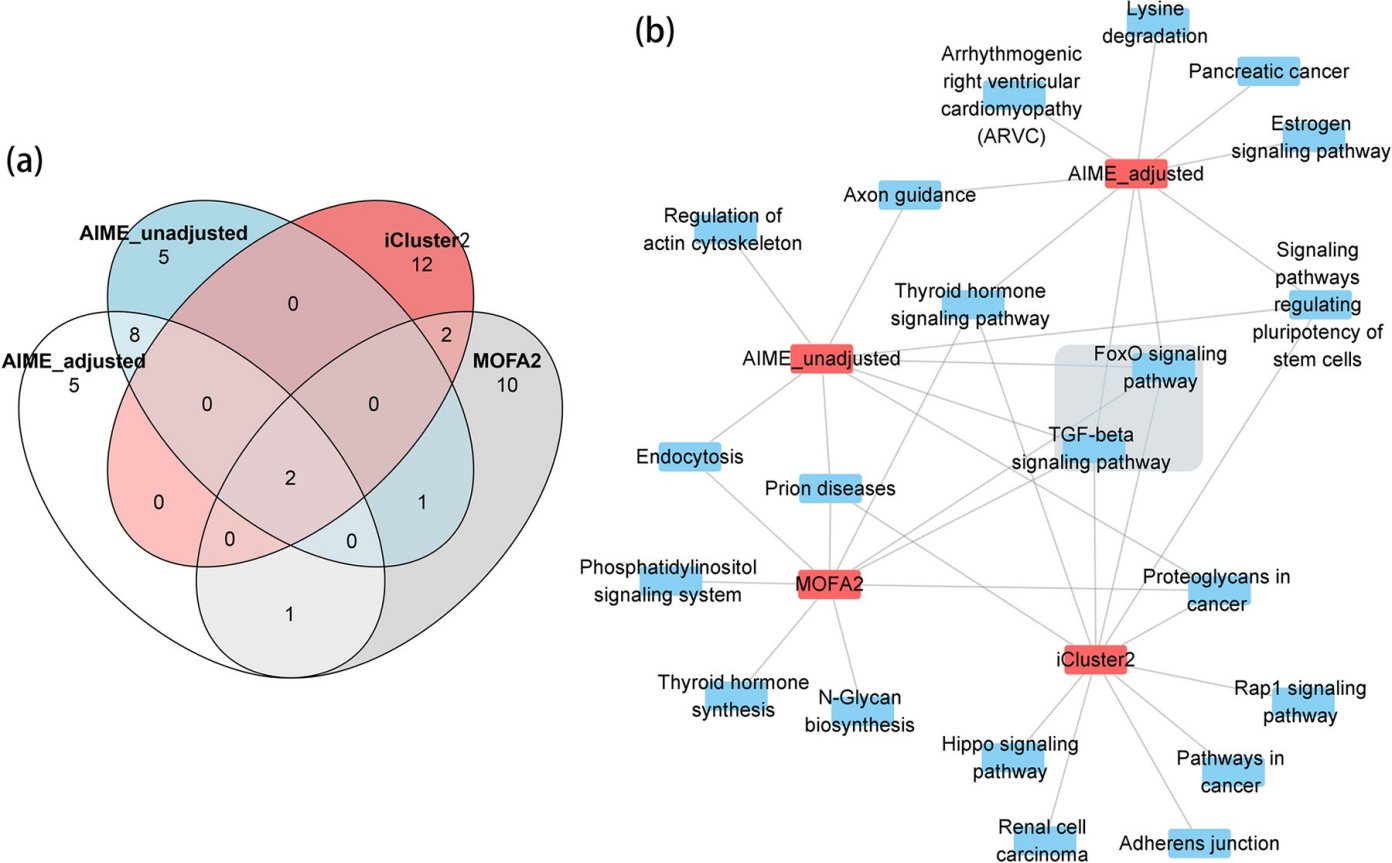
Venn Diagram of the top microRNAs and their functional analysis. (a) Venn diagrams of the top 16 microRNAs selected by AIME_adjusted (fdr≤0.1), and the top 16 microRNAs from AIME_unadjusted, MOFA2, and iCluster2. (b) The overrepresented pathways (FDR≤0.01) using miRPath 3.0.

For comparison, we also selected the top 16 miRNAs from three other analyses–AIME without adjusting for confounders, iCluster2, and MOFA2. Based on the Venn diagram, there was reasonable overlap between AIME_adjusted and AIME_unadjusted, while iCluster2 and MOFA2 tended to select different miRNAs (Fig 11A). We then examined the functionality of the top 16 contributing microRNAs by applying DIANA-miRPath v3.0 [36], followed by FDR adjustments using the Benjamini-Hochberg method. Two pathways were selected by all methods–the FoxO and TGF-beta signaling pathways (Fig 11B, grey box). AIME_adjusted selected more pathways than AIME_unadjusted. It uniquely selected important pathways including estrogen signaling pathway and pancreatic cancer. Overall, each method reflected some aspects of signaling related to breast cancer. Given the similarity in data embedding patterns, this result is expected.

**Fig 12:**
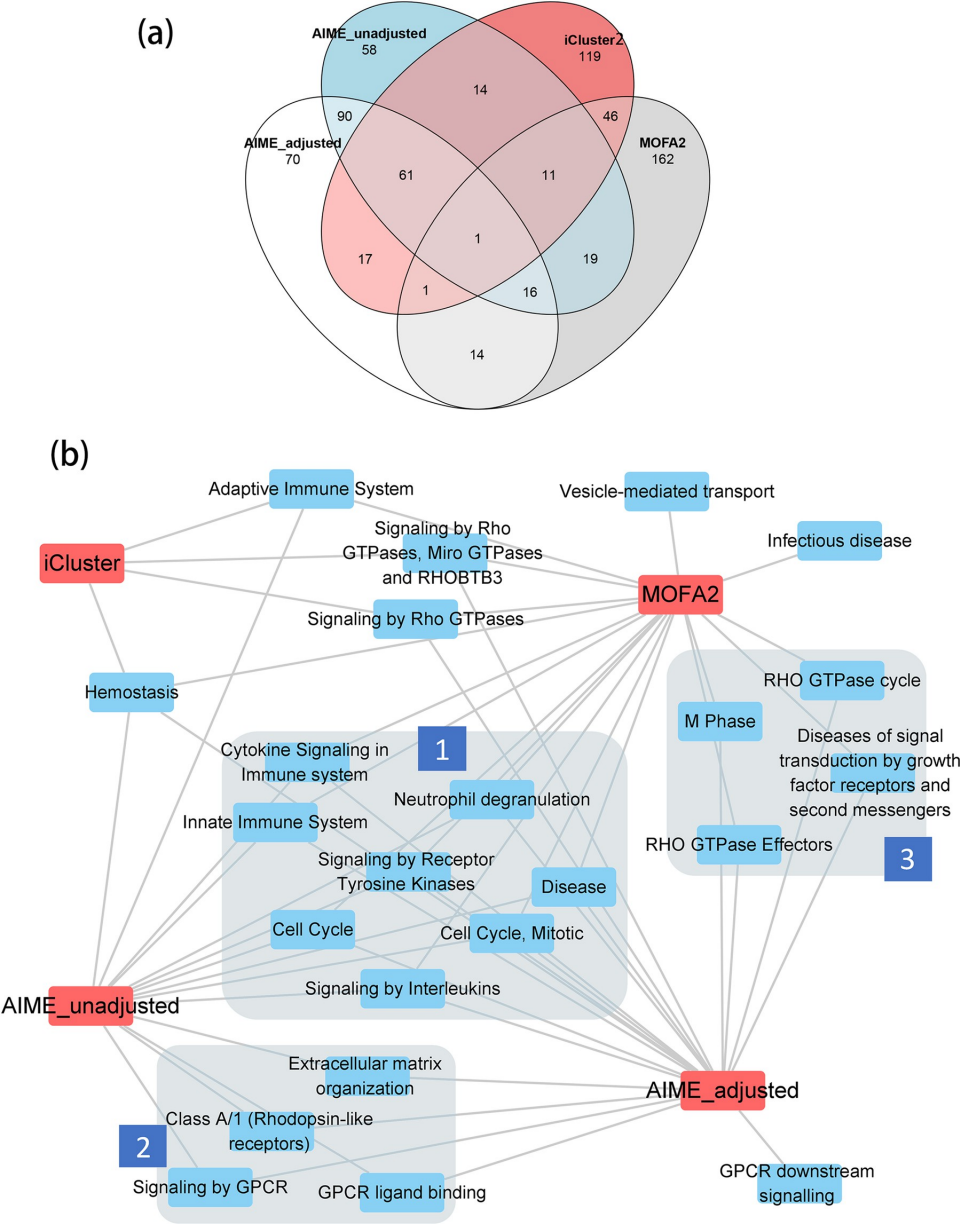
Venn Diagram of the top genes and functional analysis of the top genes. (a) Venn diagram of the top 270 genes selected by AIME_adjusted (fdr≤0.1), and the top 270 genes from AIME_unadjusted, MOFA2 and iCluster2. (b) The overrepresented pathways (FDR≤0.1) with 100~500 genes. Group 1: pathways selected by MOFA2, AIME_adjusted and AIME_unadjusted; group 2: pathways selected by AIME_adjusted and AIME_unadjusted; group 3: pathways selected by AIME_adjusted and MOFA2.

Following the same procedure described in the previous section, we selected the top 270 genes from AIME_adjusted results using the gamma distribution-based fdr approach, at fdr level of 0.1. We also selected the top 270 genes from the other three approaches: AIME_unadjusted, iCluster2, and MOFA2. Similar to the miRNA overlaps, there was reasonable overlap between AIME_adjusted andAIME_unadjusted, while iCluster2 and MOFA2 tended to select different genes than others (Fig 12A).

We then conducted gene set enrichment analysis using the fast GSEA (fgsea) package [37], which uses the ranking information of all genes. We selected reactome pathways that contain 100–500 genes, with FDR≤0.1 (Fig 12B). All methods selected the hemostasis pathway, which includes platelets, coagulation, and fibrinolysis, and is known to mediate tumor cell transformation, proliferation, and survival [39]. A group of pathways that are involved in cell cycle and immune system were selected by all methods except iCluster2 (Fig 12B, label 1). AIME_adjusted and AIME_unadjusted both selected G protein-coupled receptor pathways and extra cellular matrix organization (Fig 12B, label 2), indicating their role in cancer regulation independent of ER and T1 status. AIME_unadjusted and MOFA2 both selected RHO GTPase pathways, which are critical in wound healing and cell migration, both of which are important aspects of cancer (Fig 12B, label 3). Overall, AIME_adjusted selected the largest number of pathways, most of which have clear association with cancer.

Among the four methods in this comparison, only AIME_adjusted was able to remove the dominant effects of ER, and potentially other contributions by T1 status and age. Thus the remaining embedding patterns and the corresponding miRNA contributions were independent from ER, T1 and age. Given the fdr approach still identified significant miRNAs and miRNA-gene pairs, the detected relations were likely to be real. Indeed they point to miRNAs and biological functions that traditional methods didn’t find, validating the value of the new approach.

### 3.4. Discussions

AIME can be seen as a nonlinear equivalent to CCA, with the added capability to adjust for confounder variables. Besides being able to extract nonlinear relationships that traditional methods cannot, when sample size is large enough, AIME is even more effective than traditional linear methods such as CCA, PLS, jSVD, iCluster2 and MOFA2 in extracting linear relationships. In real data applications, AIME was able to exclude the influence of unwanted confounders and extract novel patterns. The results were easily interpretable. We believe AIME is a valuable addition to the current methods of omics data integrative analyses.

The current setup of the AIME model only allows the analysis of two data types at a time. To analyze multiple data types jointly requires major modifications to the structure of the neural network. One possible route is to put all data types in the input, reduce each data type to a lower dimension nonlinearly, and use a loss function that encourages agreement between the nonlinear embeddings. We will pursue this in future studies.

## Supporting information

S1 Fig Full simulation result. PR-AUC was used to assess each method’s success in selecting the true contributing variables.
(TIF)

S2 Fig MOFA2 results of CCLE miRNA and gene expression data. (TIF)

S3 Fig jSVD results of CCLE miRNA and gene expression data. (TIF)

S4 Fig iCluster2 results of CCLE miRNA and gene expression data. (TIF)

S5 Fig SNF results of CCLE miRNA and gene expression data. (TIF)

S6 Fig Example plots from the local fdr procedure. (a) Fitting the miRNA importance score of the CCLE data and determining the threshold. (b) Fitting the gene score for a single miRNA and determining the threshold. Red curve: estimated null component density; blue bar: selected threshold.
(TIF)

S7 Fig CCLE data: proportion of validated miRNA-gene pairs, expressed as fold-change over random pairs between any gene and the selected miRNAs at each miRNA threshold. Color curves: different fdr cutoffs to select top miRNAs.
(TIF)

S8 Fig AIME results using TCGA miRNA and gene expression data, with and without adjusting for confounders including age, T1 (tumor size) status, and estrogen receptor (ER) status. Points are colored based on ER status. Upper-right sub-plots: without adjustment for confounders; lower-left sub-plots: with adjustment for confounders.
(TIF)

S9 Fig MOFA2 results using TCGA miRNA and gene expression data. Points are colored based on PAM50 (Prosigna Breast Cancer Prognostic Gene Signature Assay) subtypes.
(TIF)

S10 Fig MOFA2 results using TCGA miRNA and gene expression data. Points are colored based on ER status.
(TIF)

S11 Fig CCA results using TCGA miRNA and gene expression data. Points are colored based on PAM50 (Prosigna Breast Cancer Prognostic Gene Signature Assay) subtypes.
(TIF)

S12 Fig CCA results using TCGA miRNA and gene expression data. Points are colored based on ER status.
(TIF)

S13 Fig jSVD results using TCGA miRNA and gene expression data. Points are colored based on PAM50 (Prosigna Breast Cancer Prognostic Gene Signature Assay) subtypes.
(TIF)

S14 Fig jSVD results using TCGA miRNA and gene expression data. Points are colored based on ER status.
(TIF)

S15 Fig iCluster2 results using TCGA miRNA and gene expression data. Points are colored based on PAM50 (Prosigna Breast Cancer Prognostic Gene Signature Assay) subtypes.
(TIF)

S16 Fig iCluster2 results using TCGA miRNA and gene expression data. Points are colored based on ER status.
(TIF)

S17 Fig SNF results using TCGA miRNA and gene expression data. Points are colored based on PAM50 (Prosigna Breast Cancer Prognostic Gene Signature Assay) subtypes.
(TIF)

S18 Fig SNF results using TCGA miRNA and gene expression data. Points are colored based on ER status.
(TIF)

S15 Fig AIME results using TCGA miRNA and gene expression data, adjusting for confounders including age and T1 (tumor size) status. Points are colored based on PAM50 (Prosigna Breast Cancer Prognostic Gene Signature Assay) subtypes.
(TIF)

S20 Fig AIME results using TCGA miRNA and gene expression data, adjusting for confounders including age and T1 (tumor size) status. Points are colored based on ER status.
(TIF)

S21 Fig AIME results using TCGA miRNA and gene expression data, adjusting for confounders including age and ER status. Points are colored based on PAM50 (Prosigna Breast Cancer Prognostic Gene Signature Assay) subtypes.
(TIF)

S22 Fig AIME results using TCGA miRNA and gene expression data, adjusting for confounders including age and ER status. Points are colored based on ER status.
(TIF)

S23 Fig BRCA data: proportion of validated miRNA-gene pairs, expressed as fold-change over random pairs between any gene and the selected miRNAs at each miRNA threshold. Color curves: different fdr cutoffs to select top miRNAs.
(TIF)
