# Supplementary figures and images for: AIME: Autoencoder-based integrative multi-omics data embedding that allows for confounder adjustments
Source: PLoS Comput Biol. 2022 Jan 26;18(1):e1009826. doi: 10.1371/journal.pcbi.1009826 (PMC8820645; doi:10.1371/journal.pcbi.1009826)

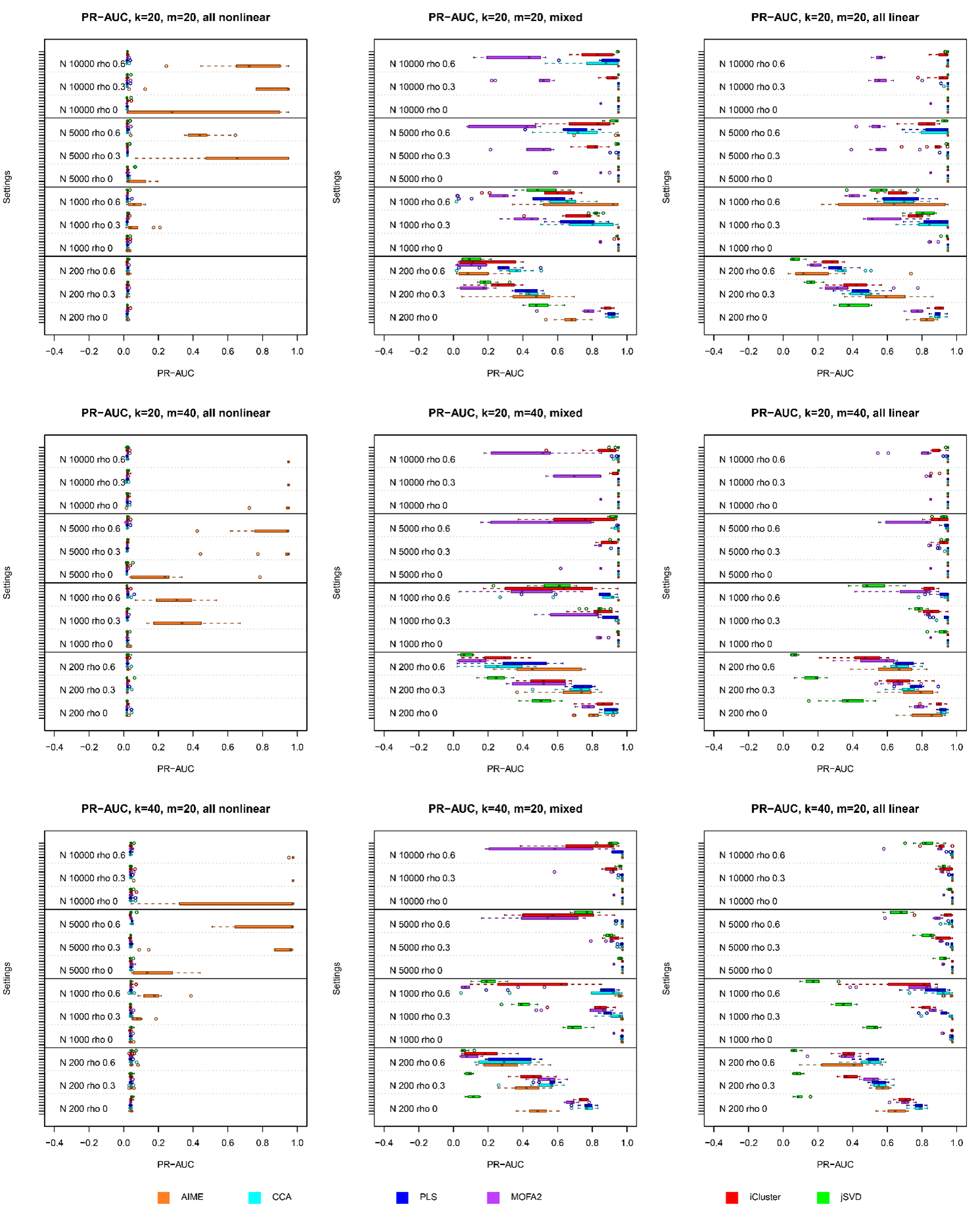

Supplement: S1 Fig — PR-AUC was used to assess each method’s success in selecting the true contributing variables. (TIF) [file pcbi.1009826.s001.tif]

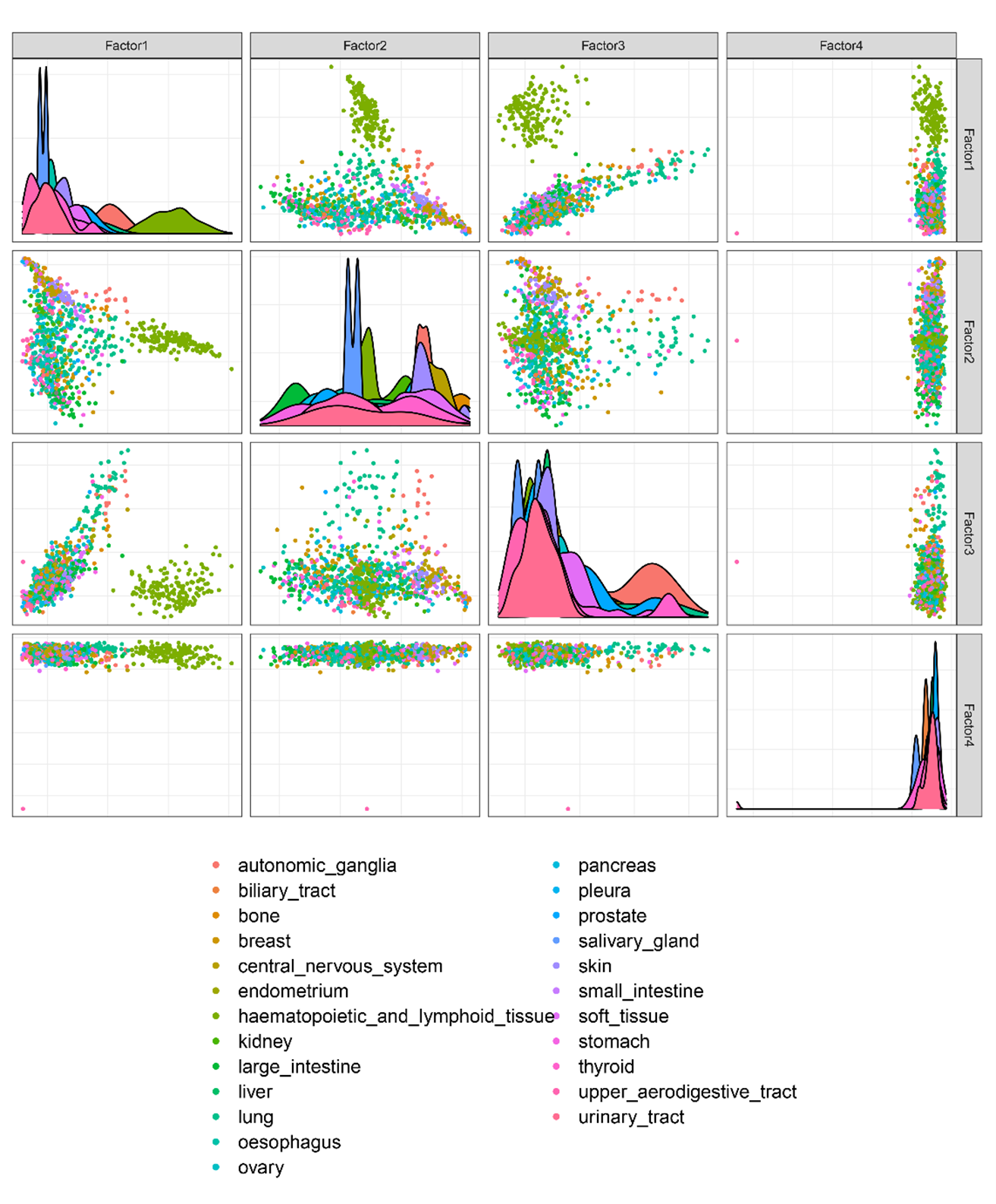

Supplement: S2 Fig — (TIF) [file pcbi.1009826.s002.tif]

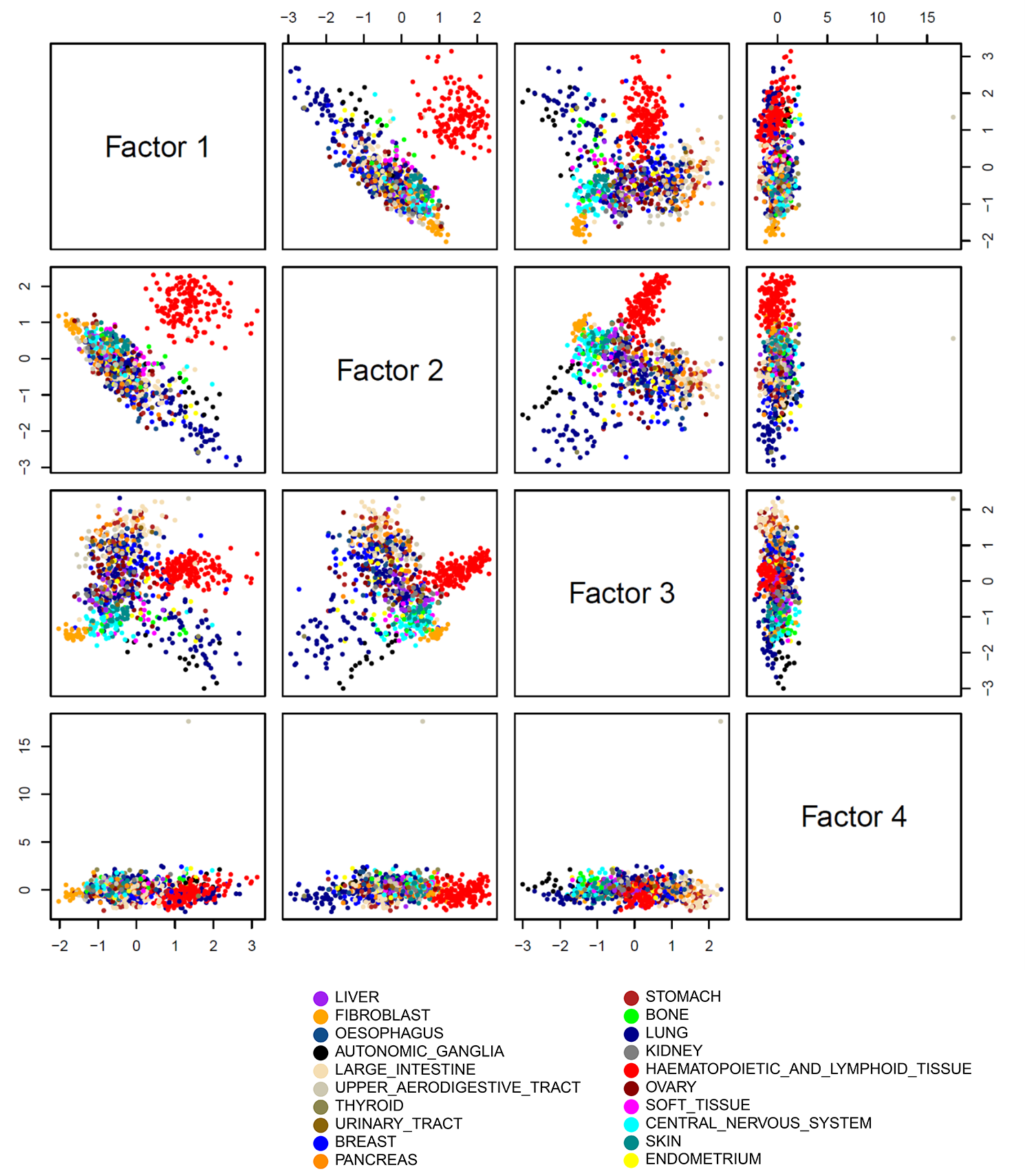

Supplement: S3 Fig — (TIF) [file pcbi.1009826.s003.tif]

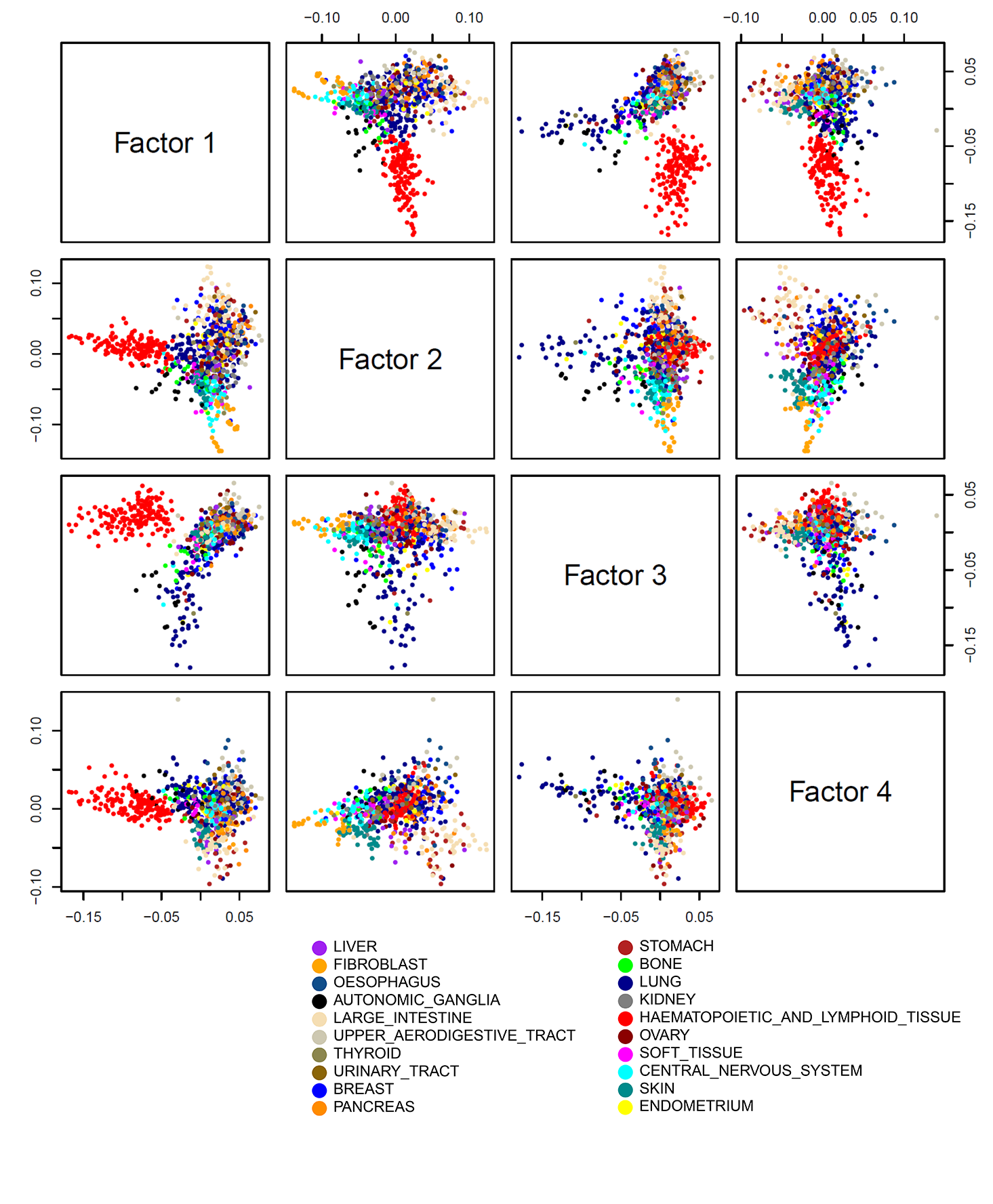

Supplement: S4 Fig — (TIF) [file pcbi.1009826.s004.tif]

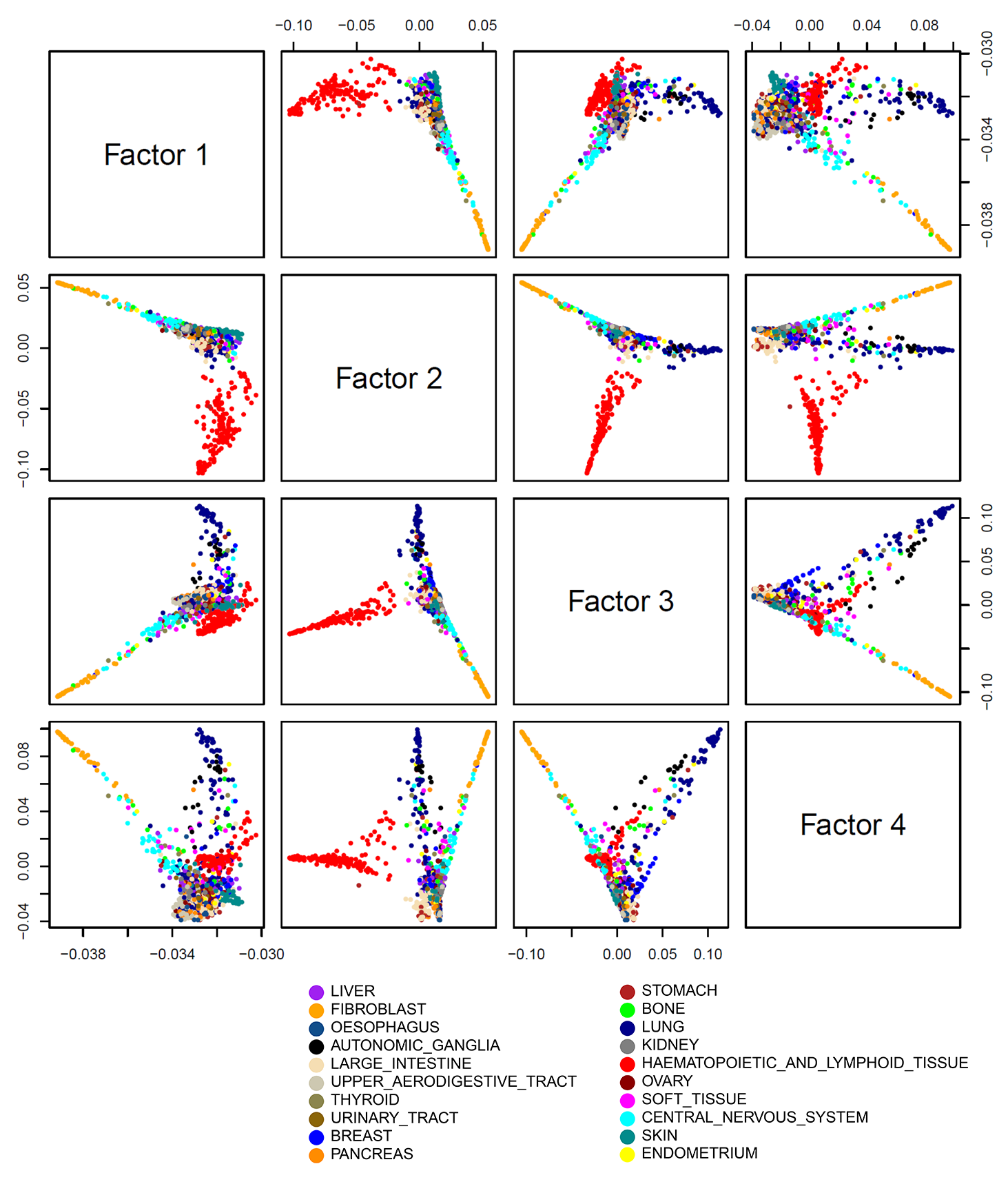

Supplement: S5 Fig — (TIF) [file pcbi.1009826.s005.tif]

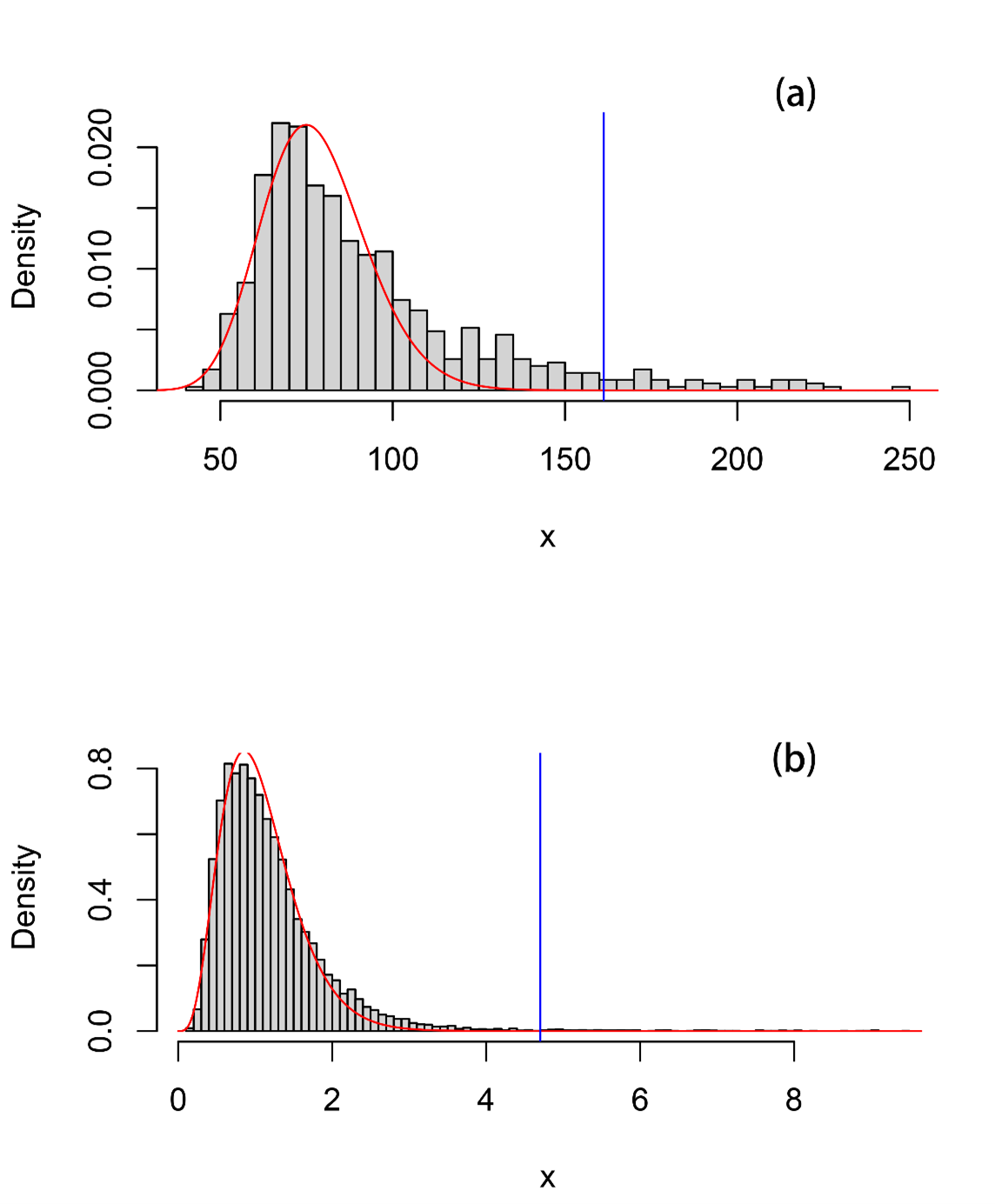

Supplement: S6 Fig — (a) Fitting the miRNA importance score of the CCLE data and determining the threshold. (b) Fitting the gene score for a single miRNA and determining the threshold. Red curve: estimated null component density; blue bar: selected threshold. (TIF) [file pcbi.1009826.s006.tif]

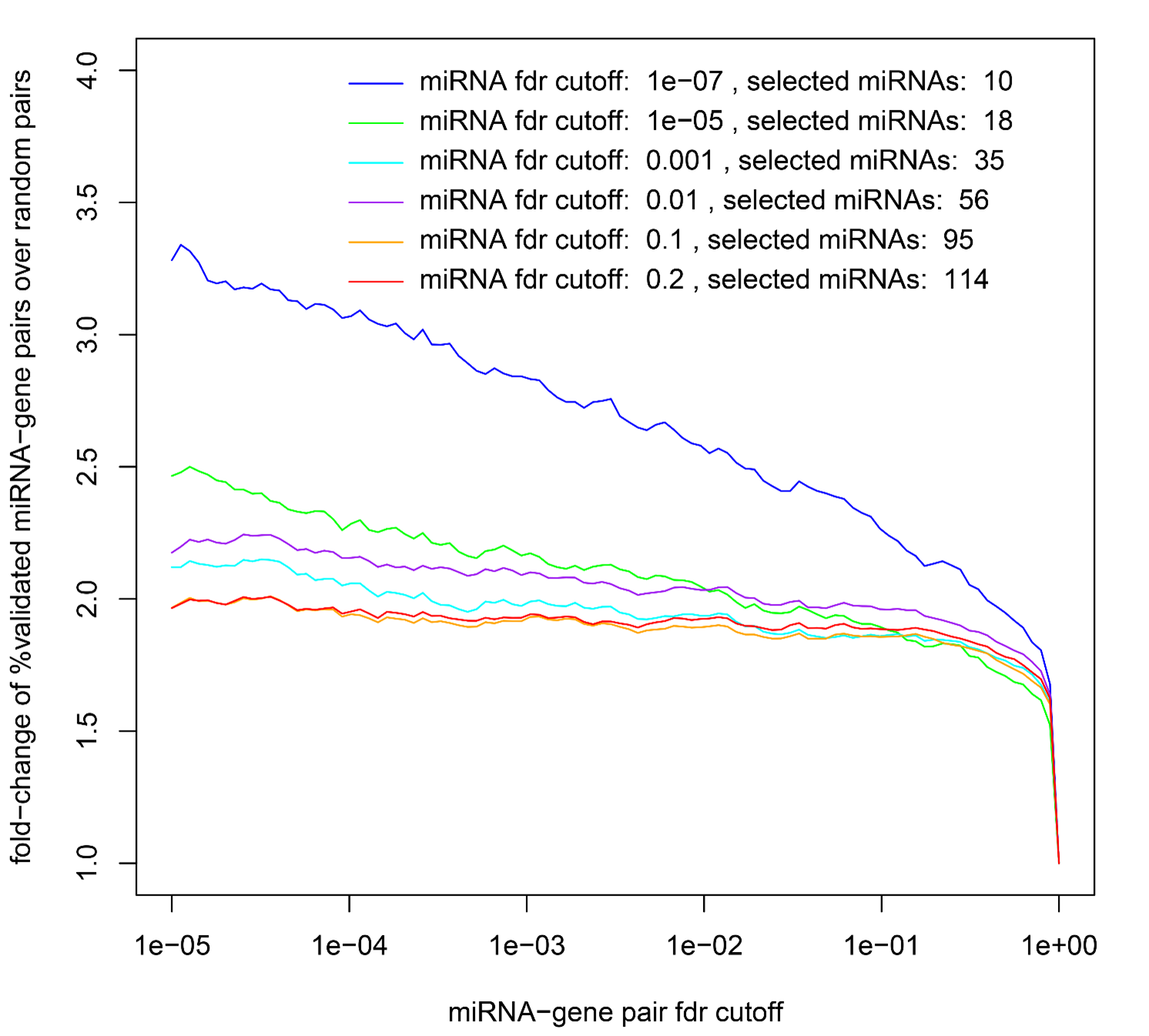

Supplement: S7 Fig — Color curves: different fdr cutoffs to select top miRNAs. (TIF) [file pcbi.1009826.s007.tif]

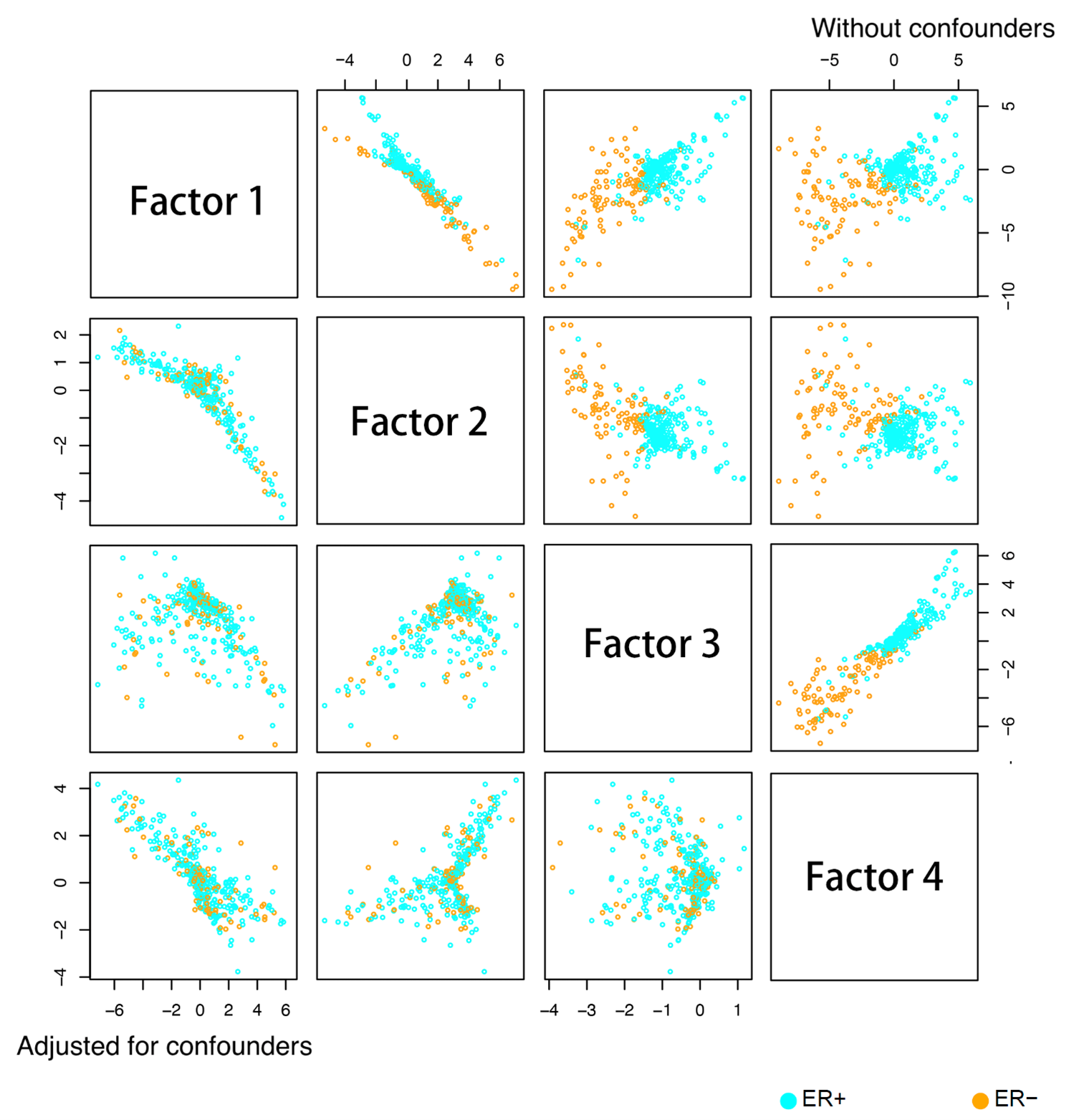

Supplement: S8 Fig — Points are colored based on ER status. Upper-right sub-plots: without adjustment for confounders; lower-left sub-plots: with adjustment for confounders. (TIF) [file pcbi.1009826.s008.tif]

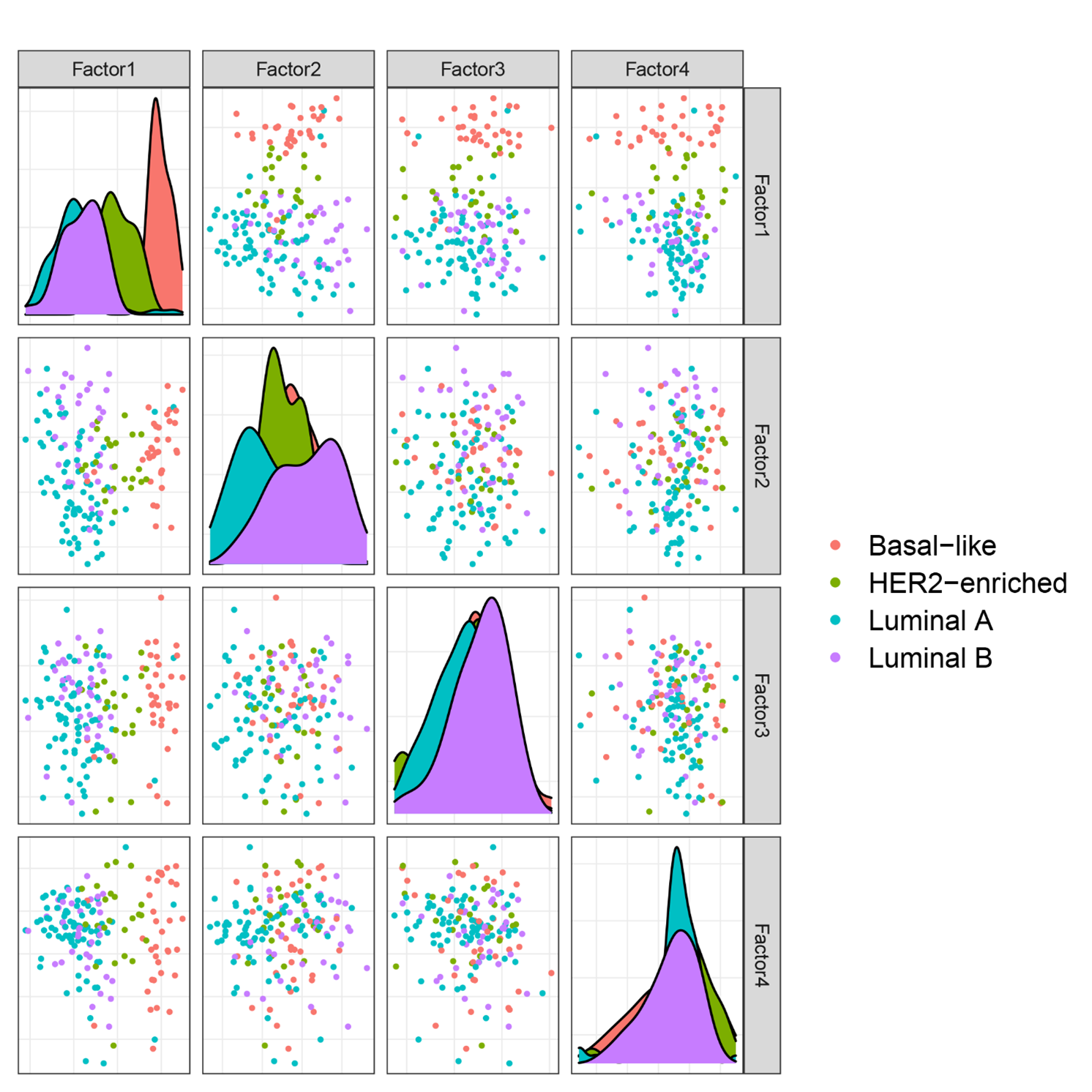

Supplement: S9 Fig — Points are colored based on PAM50 (Prosigna Breast Cancer Prognostic Gene Signature Assay) subtypes. (TIF) [file pcbi.1009826.s009.tif]

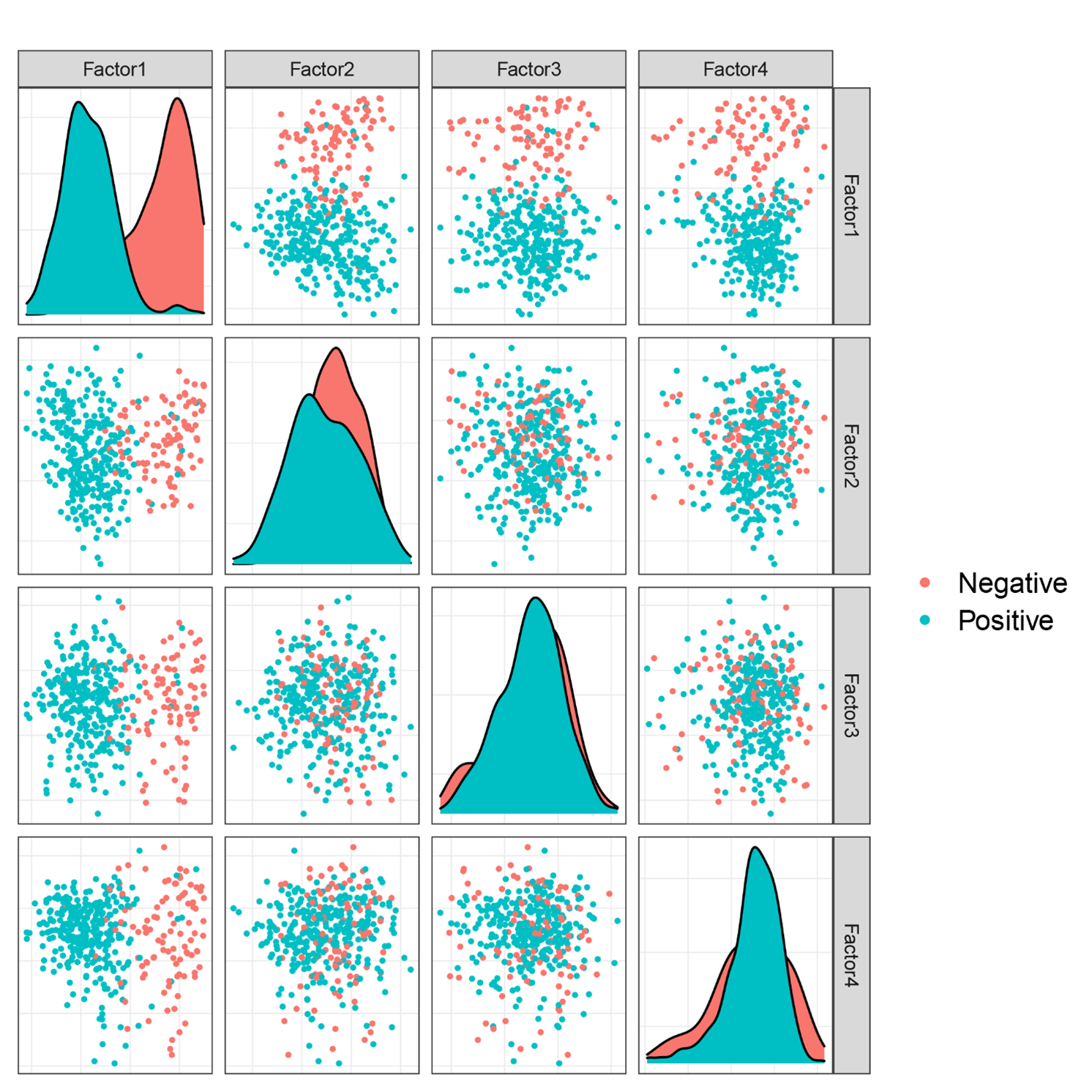

Supplement: S10 Fig — Points are colored based on ER status. (TIF) [file pcbi.1009826.s010.tif]

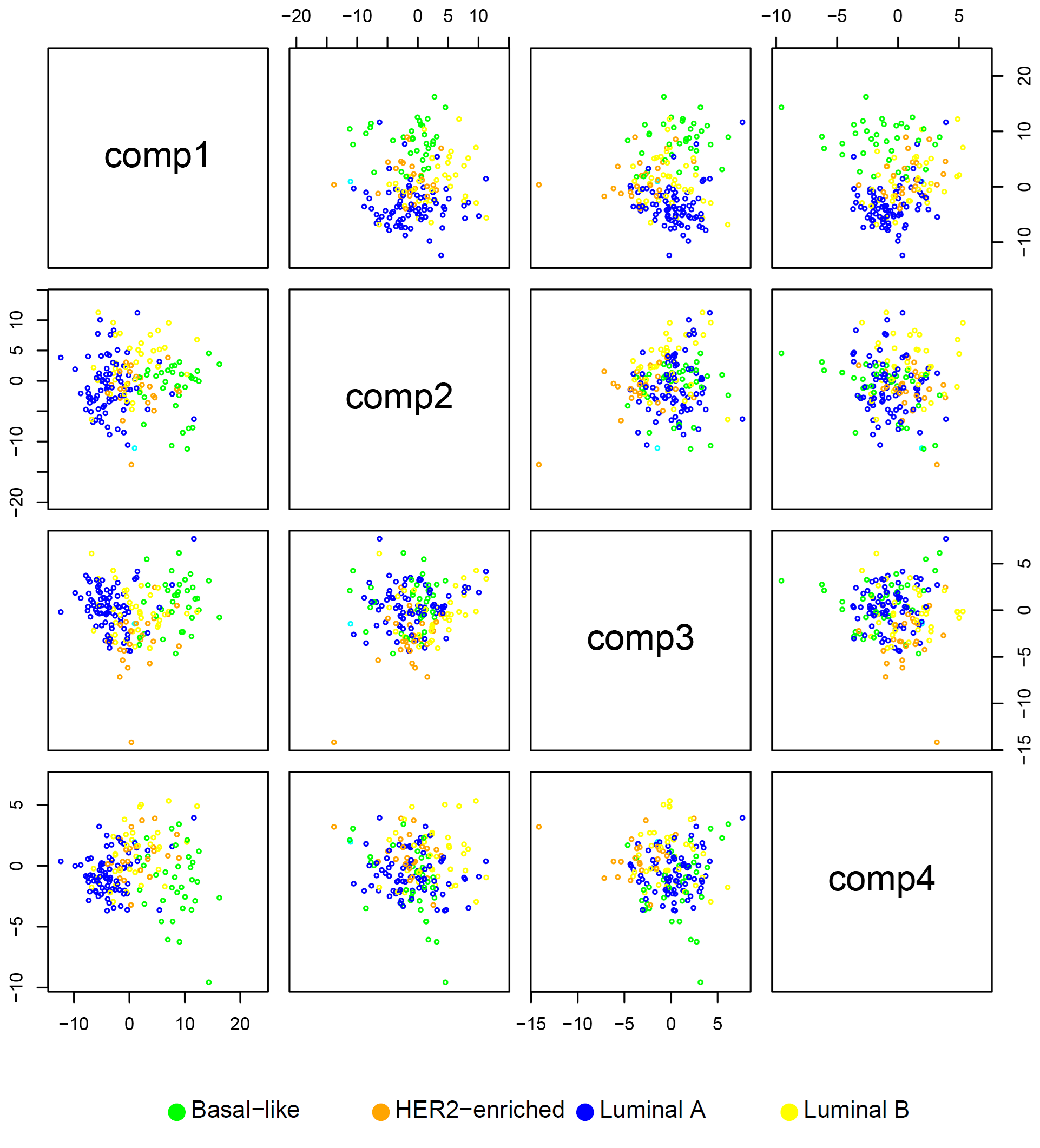

Supplement: S11 Fig — Points are colored based on PAM50 (Prosigna Breast Cancer Prognostic Gene Signature Assay) subtypes. (TIF) [file pcbi.1009826.s011.tif]

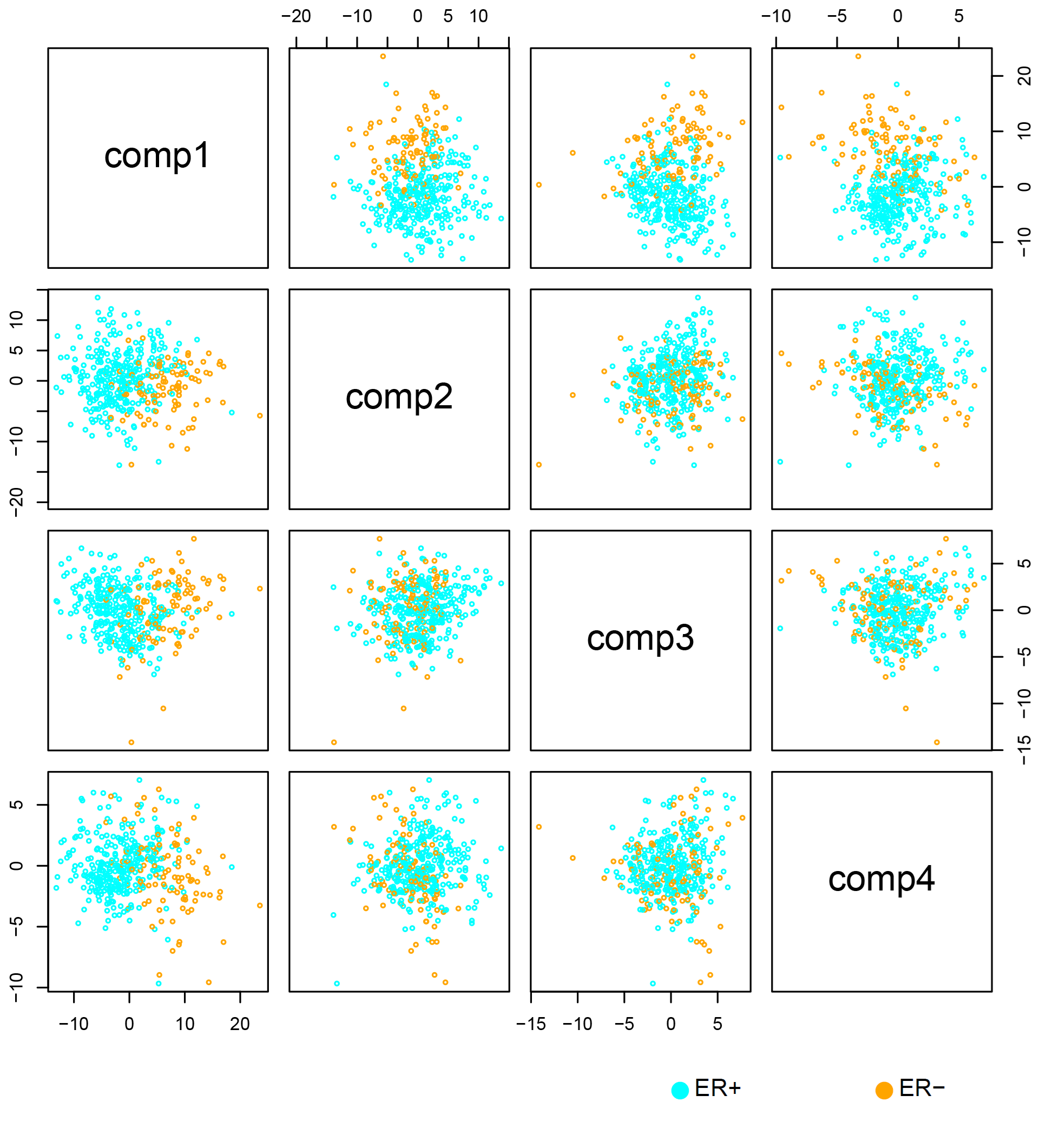

Supplement: S12 Fig — Points are colored based on ER status. (TIF) [file pcbi.1009826.s012.tif]

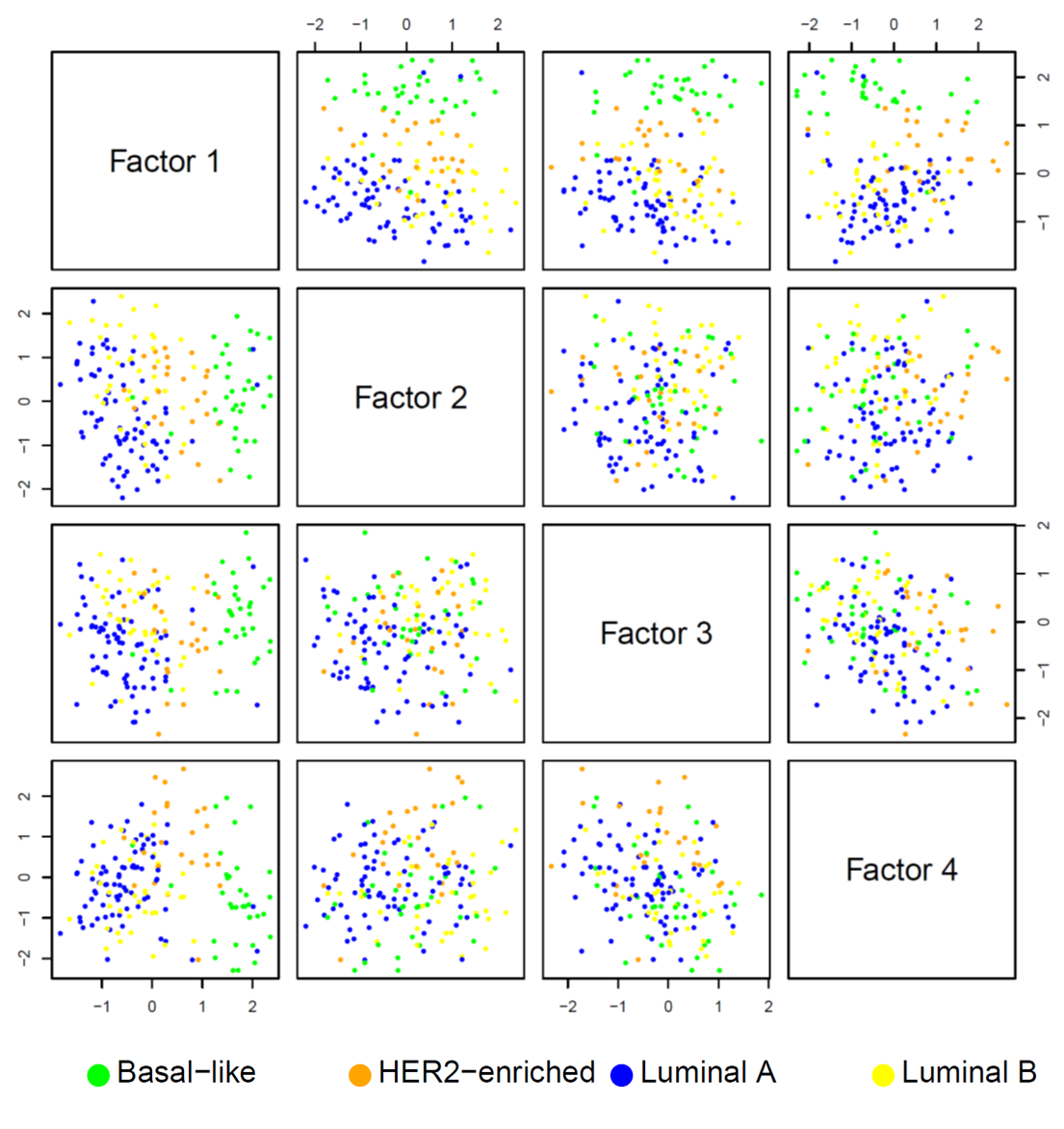

Supplement: S13 Fig — Points are colored based on PAM50 (Prosigna Breast Cancer Prognostic Gene Signature Assay) subtypes. (TIF) [file pcbi.1009826.s013.tif]

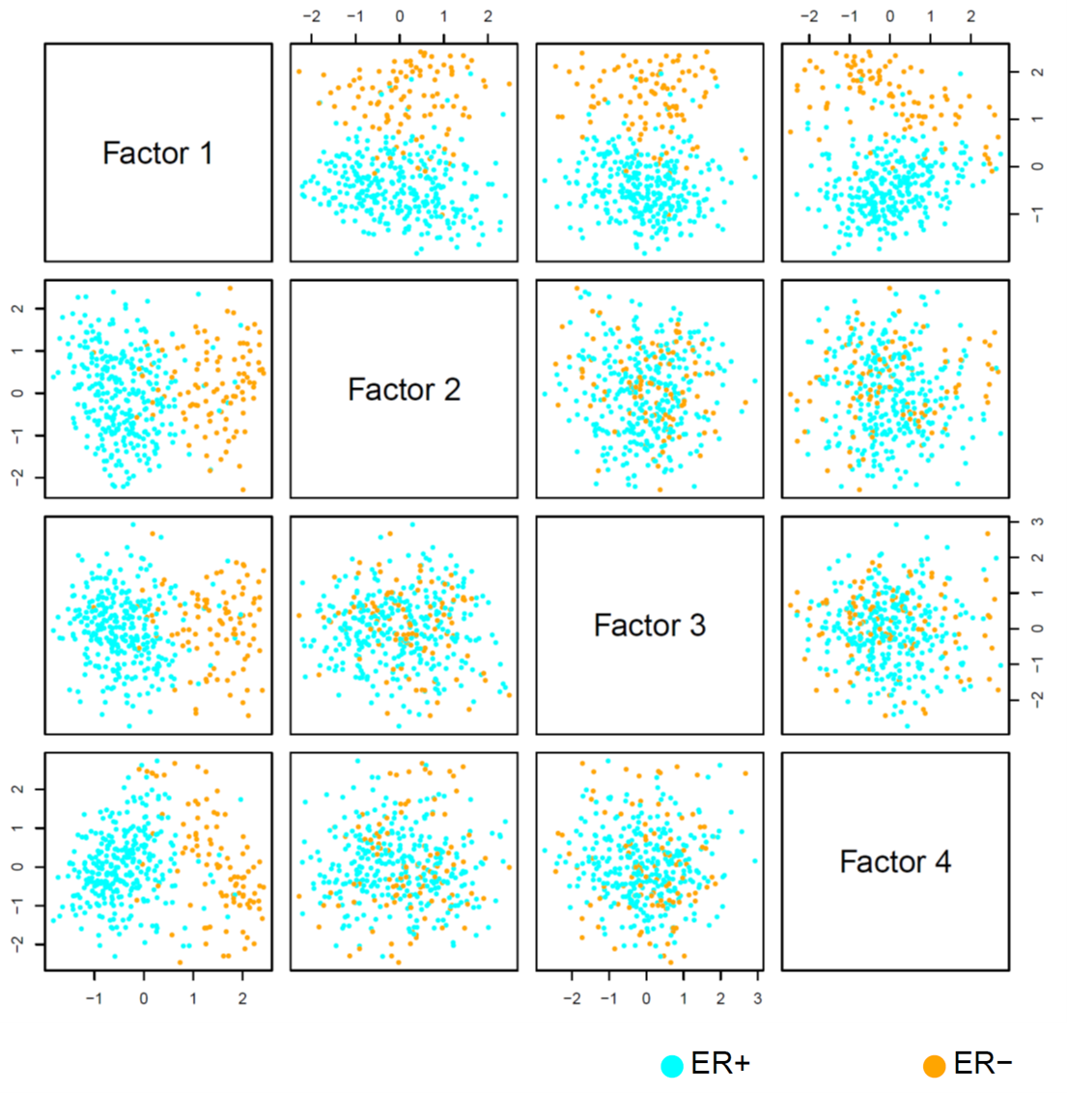

Supplement: S14 Fig — Points are colored based on ER status. (TIF) [file pcbi.1009826.s014.tif]

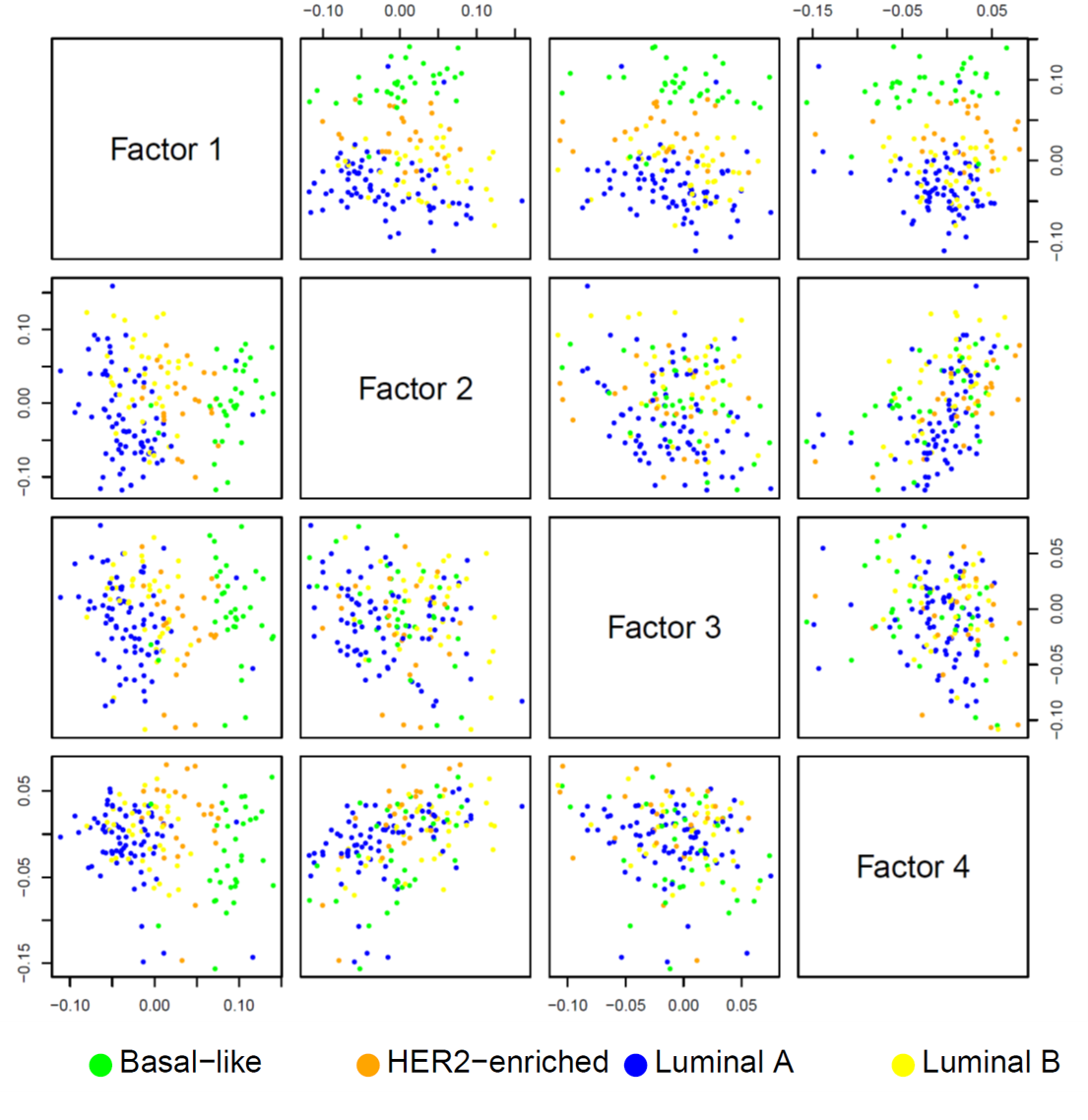

Supplement: S15 Fig — Points are colored based on PAM50 (Prosigna Breast Cancer Prognostic Gene Signature Assay) subtypes. (TIF) [file pcbi.1009826.s015.tif]

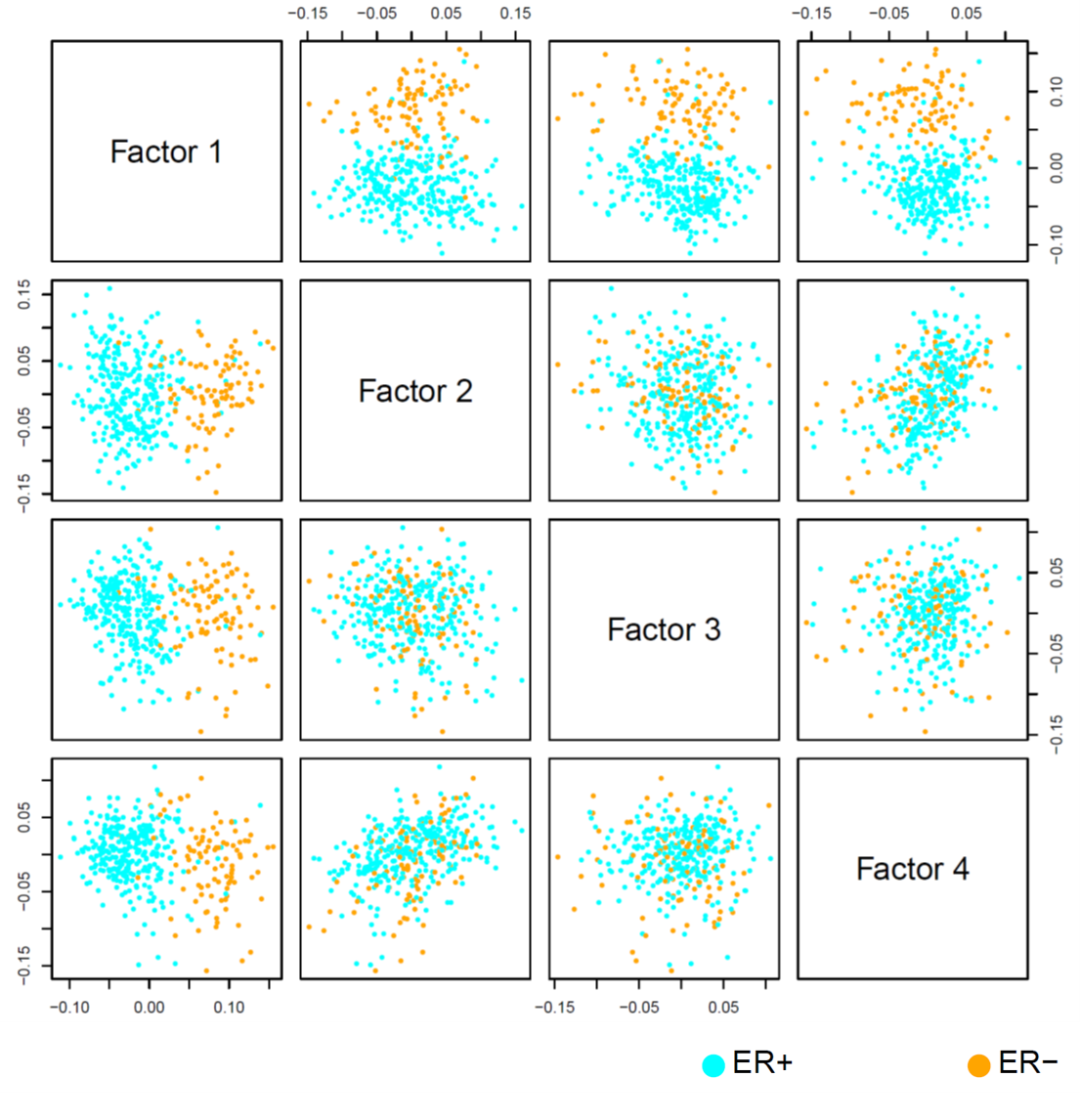

Supplement: S16 Fig — Points are colored based on ER status. (TIF) [file pcbi.1009826.s016.tif]

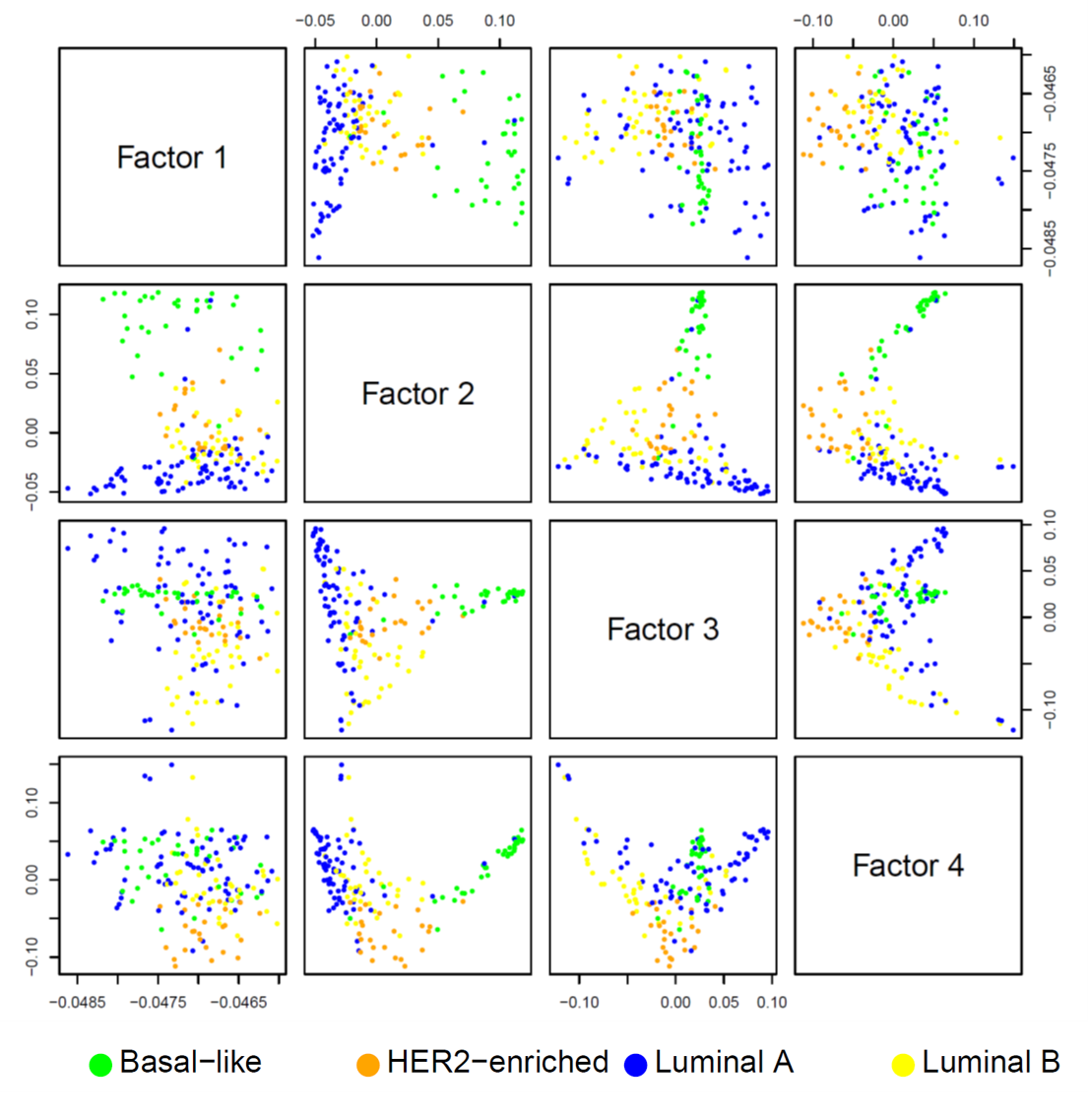

Supplement: S17 Fig — Points are colored based on PAM50 (Prosigna Breast Cancer Prognostic Gene Signature Assay) subtypes. (TIF) [file pcbi.1009826.s017.tif]

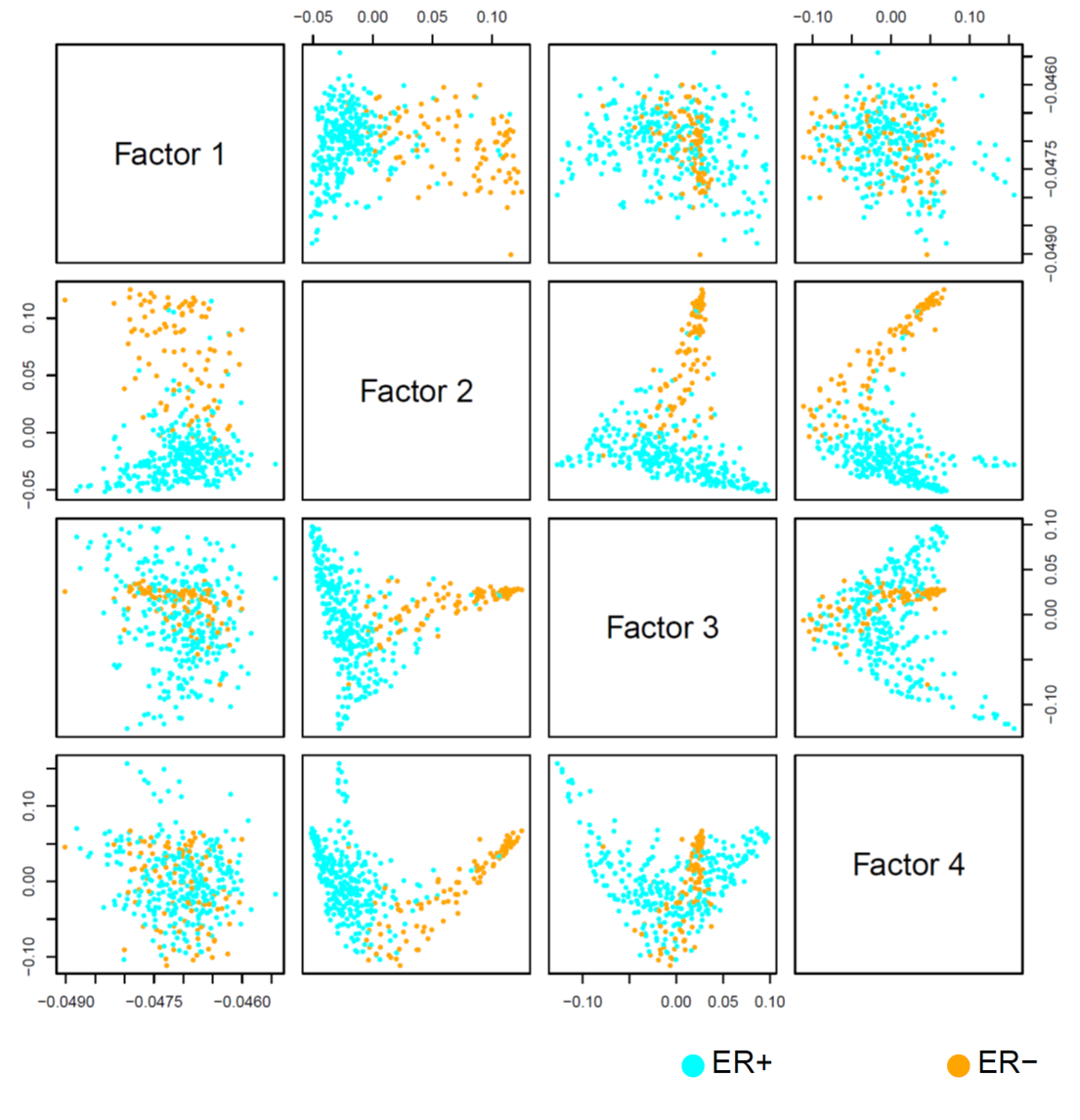

Supplement: S18 Fig — Points are colored based on ER status. (TIF) [file pcbi.1009826.s018.tif]

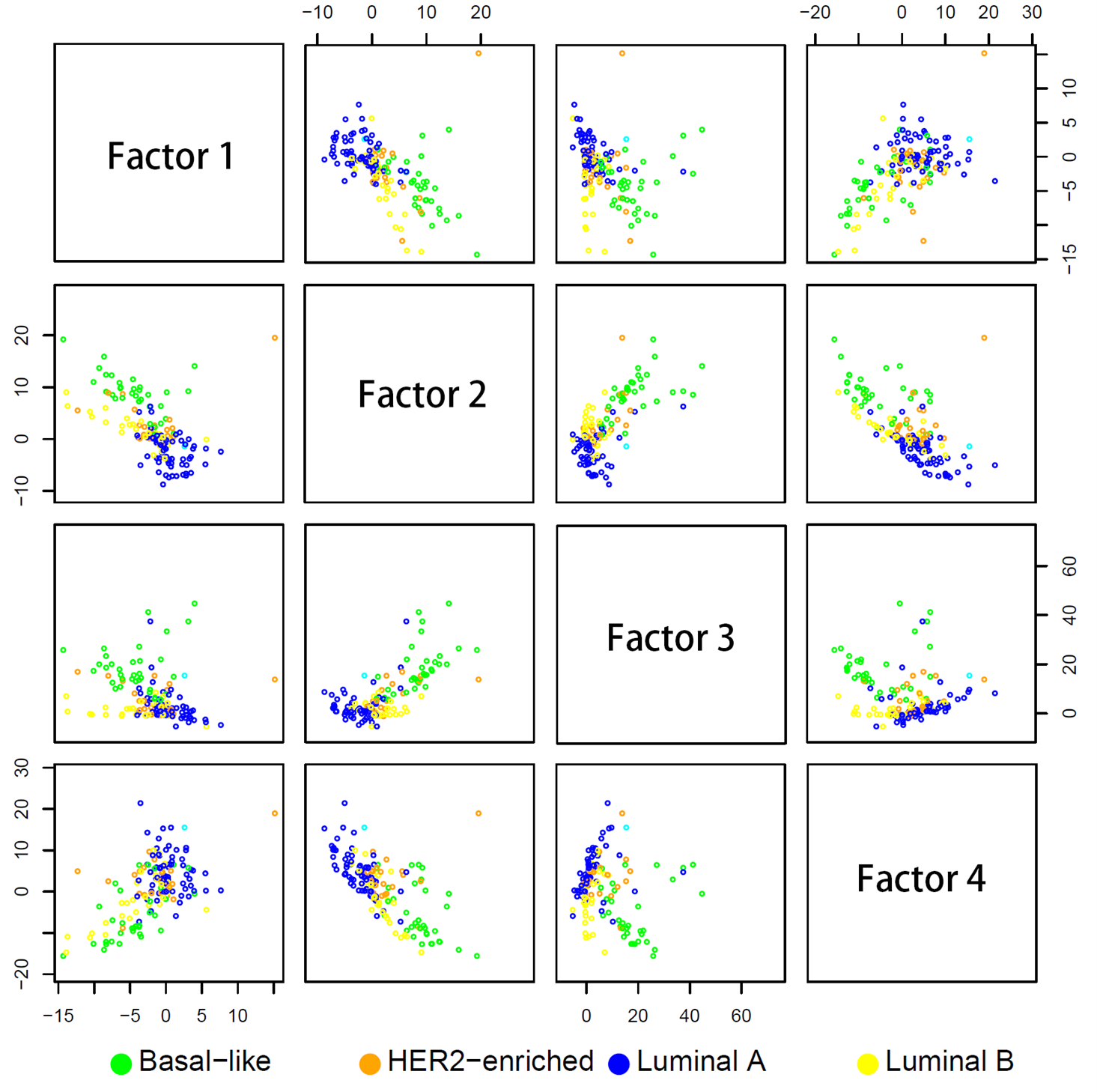

Supplement: S15 Fig — Points are colored based on PAM50 (Prosigna Breast Cancer Prognostic Gene Signature Assay) subtypes. (TIF) [file pcbi.1009826.s019.tif]

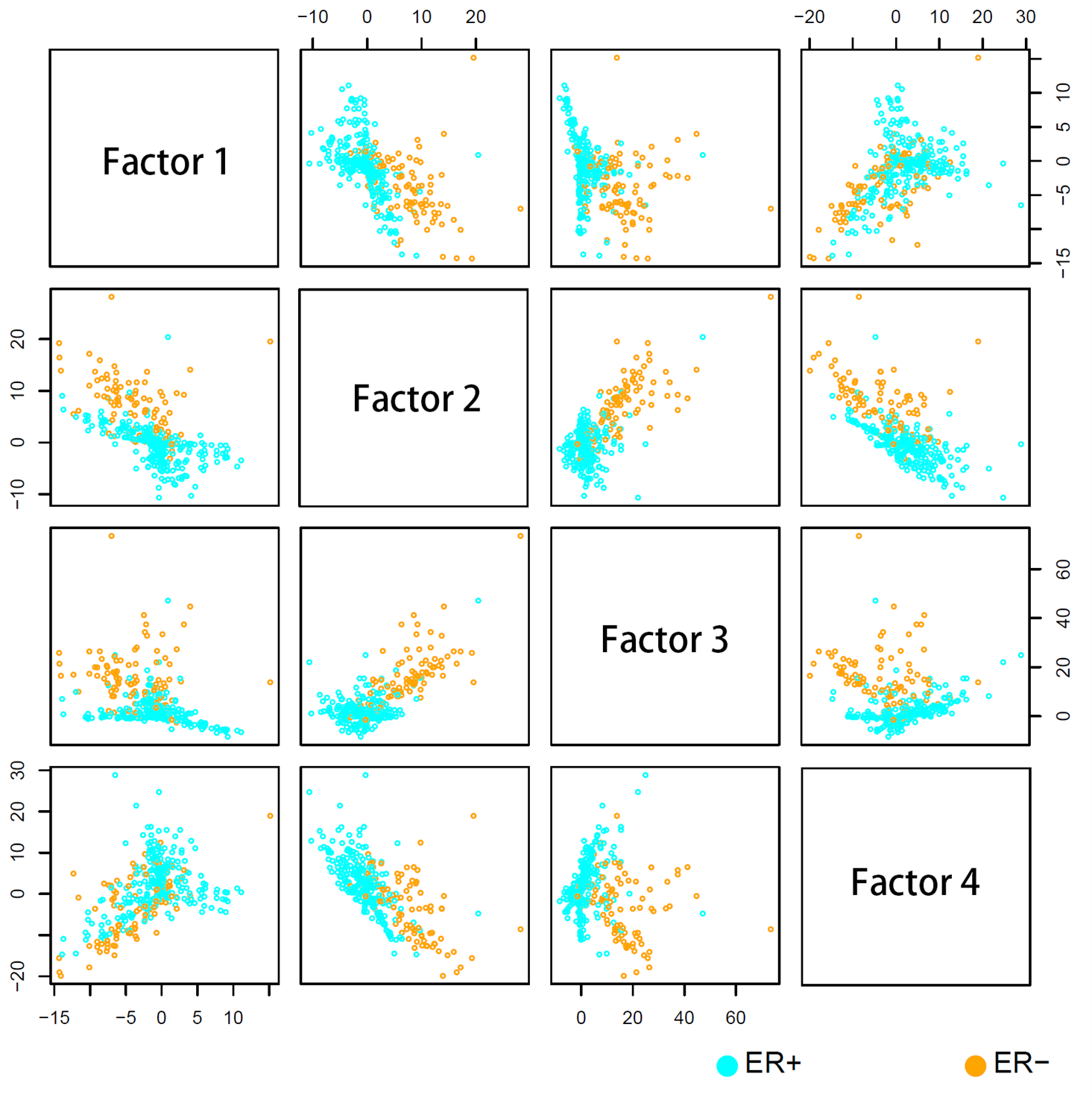

Supplement: S20 Fig — Points are colored based on ER status. (TIF) [file pcbi.1009826.s020.tif]

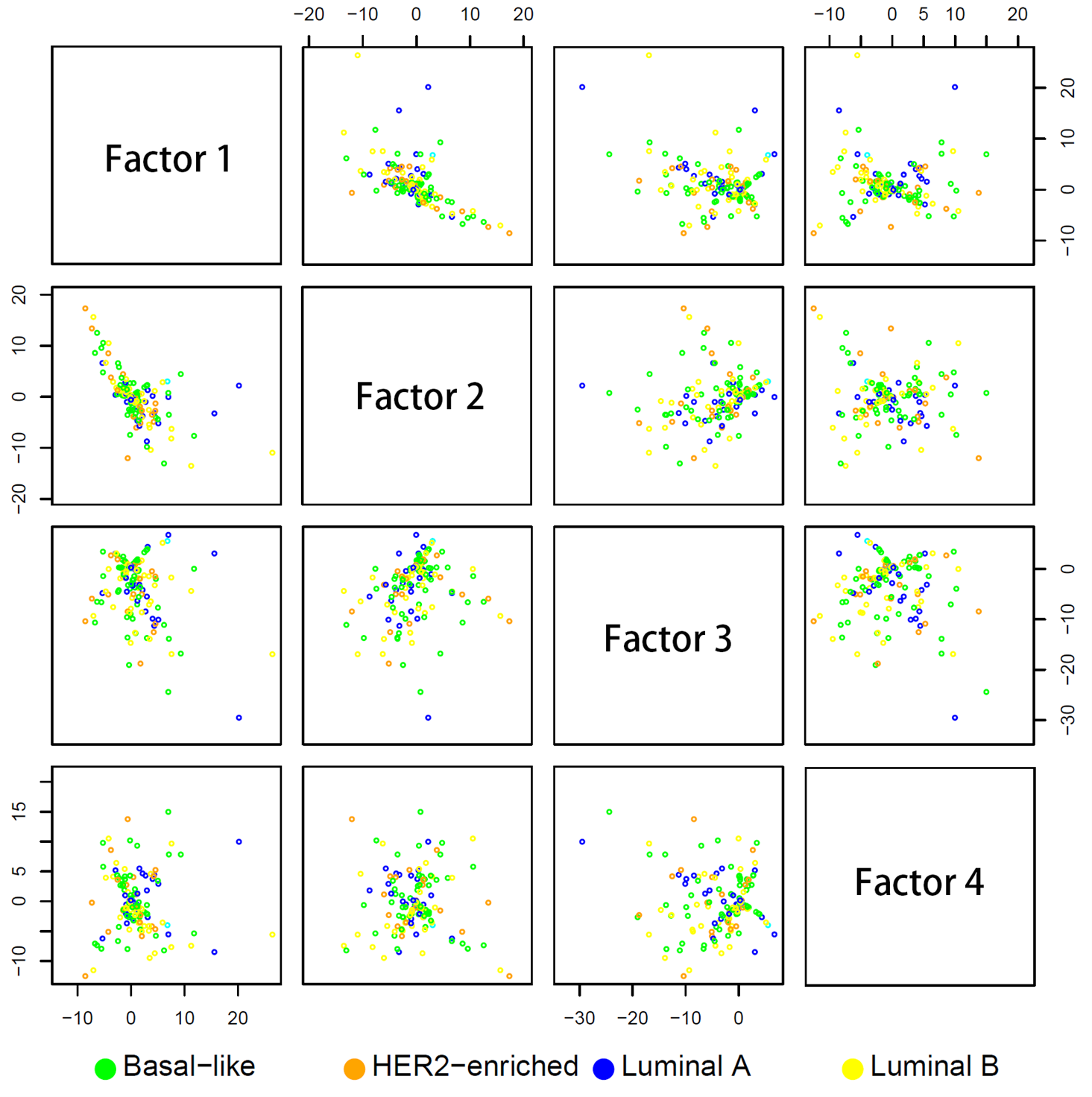

Supplement: S21 Fig — Points are colored based on PAM50 (Prosigna Breast Cancer Prognostic Gene Signature Assay) subtypes. (TIF) [file pcbi.1009826.s021.tif]

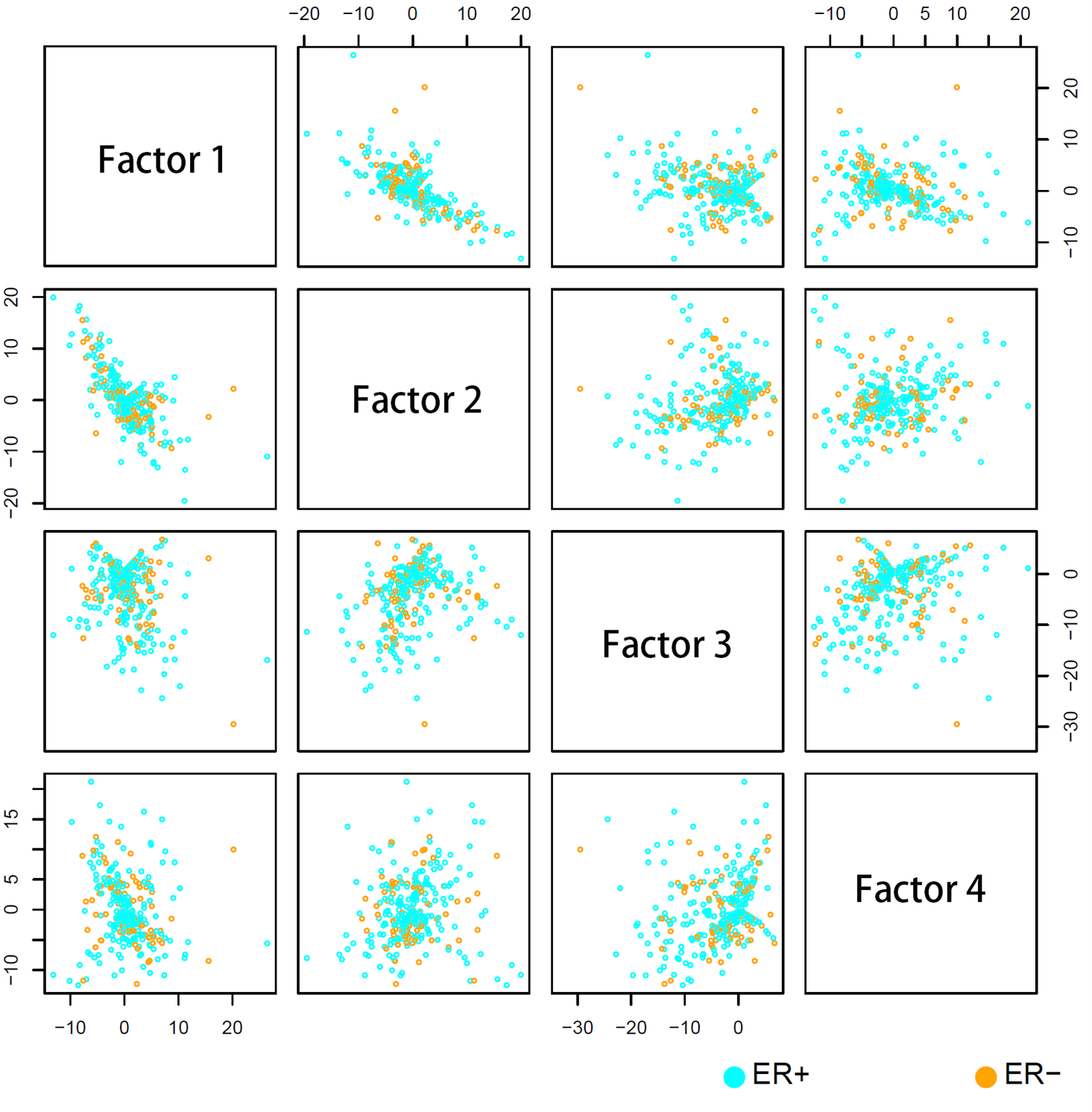

Supplement: S22 Fig — Points are colored based on ER status. (TIF) [file pcbi.1009826.s022.tif]

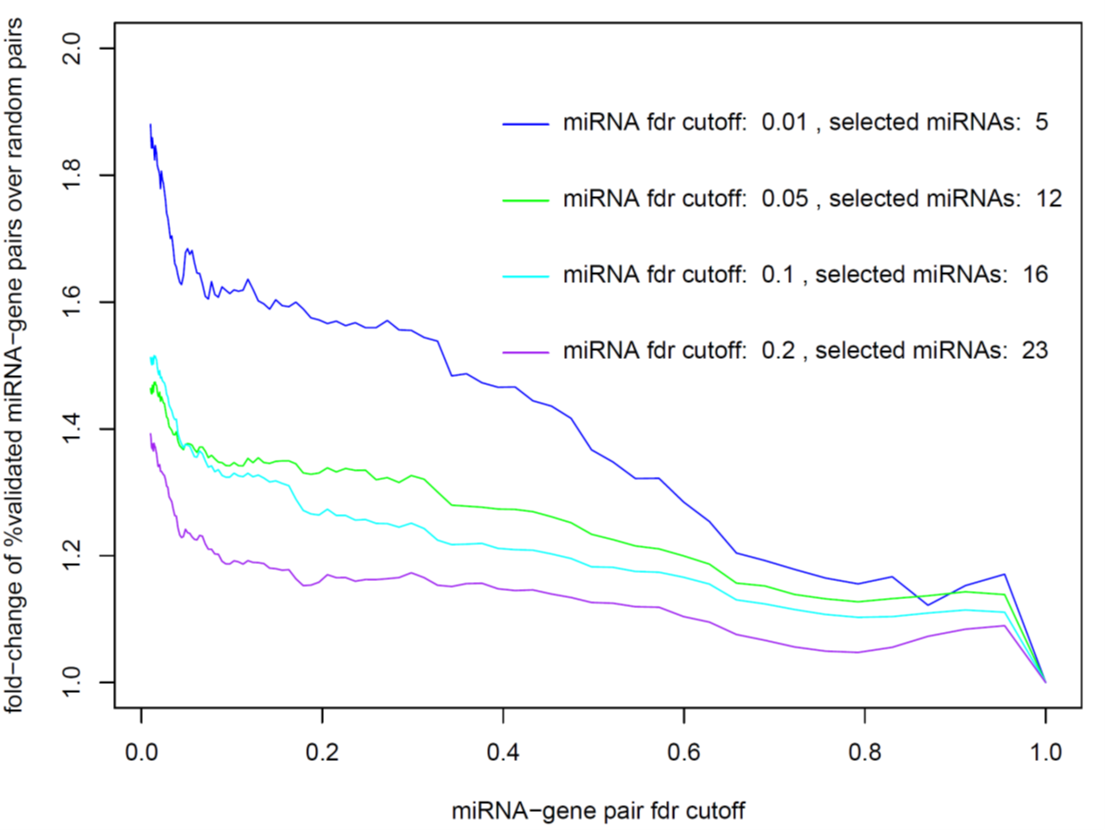

Supplement: S23 Fig — Color curves: different fdr cutoffs to select top miRNAs. (TIF) [file pcbi.1009826.s023.tif]
